# Fluorescence Sensing Technologies for Ophthalmic Diagnosis

**DOI:** 10.1021/acssensors.2c00313

**Published:** 2022-05-31

**Authors:** Yuqi Shi, Yubing Hu, Nan Jiang, Ali K. Yetisen

**Affiliations:** † Department of Chemical Engineering, Imperial College London, South Kensington, London, SW7 2BU, United Kingdom; ‡ West China School of Basic Medical Sciences and Forensic Medicine, Sichuan University, Chengdu 610041, China

**Keywords:** Fluorescence biosensing, Ophthalmological diagnostics, Fluorescence imaging, Contact lens sensors, Point-of-Care, Tear monitoring, Fluorescent tear diagnosis, Smartphone readout devices, Ocular diseases

## Abstract

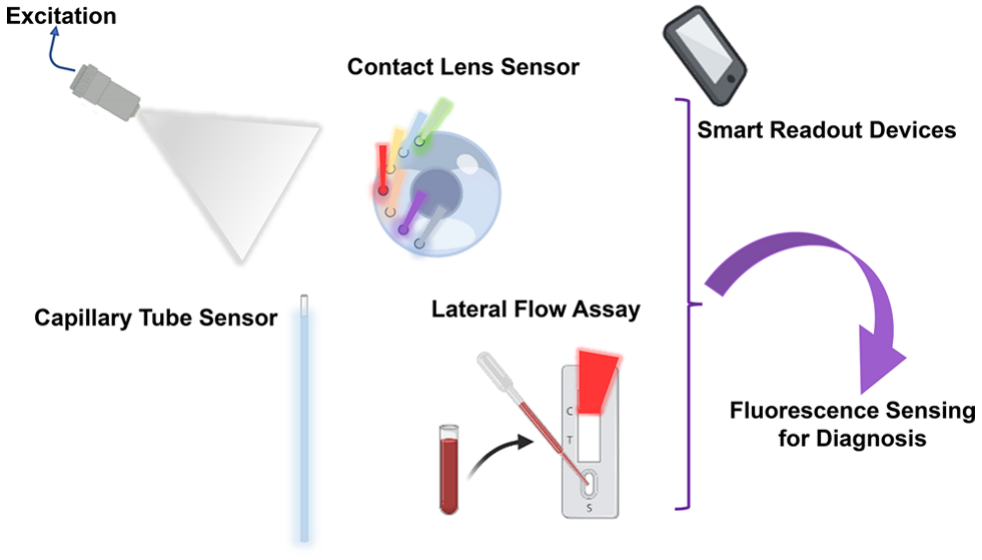

Personalized and point-of-care (POC) diagnoses are critical for ocular physiology and disease diagnosis. Real-time monitoring and continuous sampling abilities of tear fluid and user-friendliness have become the key characteristics for the applied ophthalmic techniques. Fluorescence technologies, as one of the most popular methods that can fulfill the requirements of clinical ophthalmic applications for optical sensing, have been raised and applied for tear sensing and diagnostic platforms in recent decades. Wearable sensors in this case have been increasingly developed for ocular diagnosis. Contact lenses, as one of the commercialized and popular tools for ocular dysfunction, have been developed as a platform for fluorescence sensing in tears diagnostics and real-time monitoring. Numbers of biochemical analytes have been examined through developed fluorescent contact lens sensors, including pH values, electrolytes, glucose, and enzymes. These sensors have been proven for monitoring ocular conditions, enhancing and detecting medical treatments, and tracking efficiency of related ophthalmic surgeries at POC settings. This review summarizes the applied ophthalmic fluorescence sensing technologies in tears for ocular diagnosis and monitoring. In addition, the cooperation of fabricated fluorescent sensor with mobile phone readout devices for diagnosing ocular diseases with specific biomarkers continuously is also discussed. Further perspectives for the developments and applications of fluorescent ocular sensing and diagnosing technologies are also provided.

As the second most complicated and important part of the human body, the human eye is responsible for receiving, transforming, transmitting, and assimilating informative messages.^1^ Ocular defects have affected more than 2.2 billion people around the world with different severities of visual dysfunction based on the analytical results from the World Health Organization (WHO).^2−4^ Thirty-six million people from this investigation are blind and over 50; moreover, the majority of these patients with ocular dysfunctions are from developing countries.^3^ Visual impairment is usually caused by different common ocular diseases, including refractive errors, corneal opacification, age-related macular degeneration (AMD), trachoma, glaucoma, cataracts, diabetic retinopathy, and some undetermined diseases (Figure 1). Blindness and visual impairment can be the worst influence of these diseases and are accompanied by other functional impairments, such as intellectual disability, cerebral palsy, and epilepsy.^4,5^ Moreover, the total expenditure of ophthalmic health care costs has reached around $3 trillion,^5^ and the prescription drug expenditure was reported to be over $1 billion during 2013.^6^ In addition, the largest direct medical cost is the hospitalization medical services for diagnosing and treating vision impairment and blindness at the primary phase.^7^ In recent decades, ocular therapeutic treatments and diagnosis have been highly developed and applied for personalized treatment, and some therapeutic technologies have been applied for real-time ophthalmic monitoring. Ophthalmologic technologies have been improved from both clinical trials and fundamental laboratory-based research. Current clinical ophthalmic spectroscopic applications contain both routine assessment photographic technologies and ocular segment imaging technologies. Routine detection of ocular disease diagnosis consists of retinal scopes,^8^ external eye photography in cooperation with a hand-held smartphone,^9^ and gonioscopy technologies.^10,11^ Further developments for spectroscopic assessment of ocular diseases have been established during the recent two decades and utilized for ophthalmologists clinically for real-time and dynamic monitoring, such as optical coherence tomography (OCT),^12−19^ confocal scanning laser ophthalmoscope (cSLO),^20−22^ scanning laser polarimeter (GDx),^23^ and fundus autofluorescence imaging.^24^ Several ophthalmic technologies have also been applied with the hand-held spectroscopic devices at point-of-care (POC) settings in recent years, such as digital cameras and smartphone devices, and are treated as one of the emerging clinical applications in ophthalmology for ocular imaging diagnosis and monitoring.^9^

**Figure 1:**
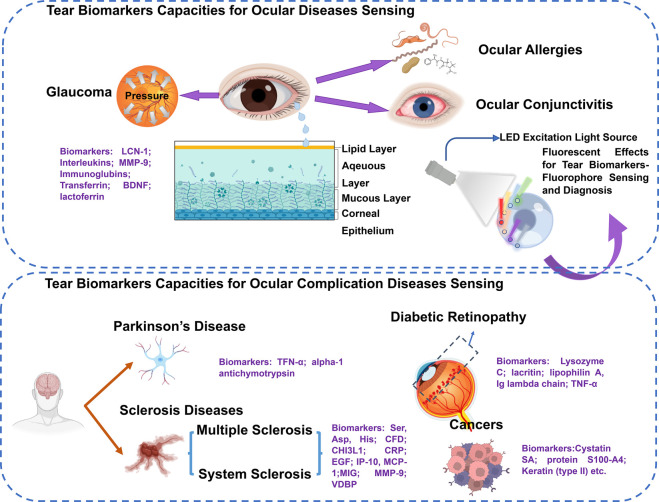
Summary of tear capacities for detection of various biomarkers in ocular diseases (top) and ocular complication disease fluorescent sensing (bottom). Biomarkers in purple are potential indicators for specific ocular diseases and ocular-related diseases, such as cancer, Parkinson’s disease, sclerosis disease, and diabetic retinopathy.^30,62−77^ Fluorescent sensing technologies can be applied for tear biomarker sensing aiming to indicate and monitor related ocular disease to achieve personal and POC diagnosis.

Sensing of tears for diagnosing and monitoring ophthalmic diseases has been emerging in the past decade.^25,26^ There are three layers within tear fluid, including the outer lipid layer, the aqueous layer, and the mucous layer (Figure 1).^27^ As tears obtain similar but simple compositions compared to blood, their potential for diagnosing various diseases has been evaluated. Mitochondrial energy metabolism and some other specific metabolic processes occur during plasma leakage and lead to transference of the components from the blood through the barrier to tears.^27,28^ Hence, tear fluid has a wide potential detection range for developing innovative diagnostic platforms of ocular diseases and other developmental ocular dysfunctions, such as cancer, neurological disorders, and diabetes.^29−34^ A large number of analytes within the tear fluid have been examined and treated as potential biomarkers for diagnosing and monitoring various ophthalmic diseases, including ocular diseases and ocular complication diseases (Figure 1).^35−46^ Therefore, the cooperation of specific technologies for target biomarkers in tear fluid provides more possibilities to identify the pathophysiology of ocular diseases. Instead of direct biomarker monitoring and detection within tears, other evaluation criteria have also been studied for ocular disease diagnosis such as moisture content and intraocular pressure.^47,48^ These developed sensors for physical signals commonly collaborate with the contact lens-based detection and aim especially for dry eye diseases and glaucoma examinations. There have been various biosensors fabricated for tear analyte detection in ophthalmological diagnosis on either paper-based or contact lens-based platforms during the past decade, including colorimetric,^25,49−51^ fluorescent,^52−55^ electrochemical,^56−60^ and photonic crystal sensing.^61^

Fluorescence sensing of biomarkers in tears is remarkable, especially for glucose,^78^ pH, and electrolytes.^52^ Performance of different fluorescent tear sensors (especially contact lens type of sensor) varies depending on different status, the range of detection for biomarkers, and the response time of the sensor, for instance. Fluorescence sensors have always been treated for various scientific applications, such as fluorescent labeling, biological detections, mineralogy, gemmology, and cosmic-ray detection.^79^ Based on the advantages of high sensitivity, specificity, easy operation, and low cost, fluorescence sensors have emerged in recent decades. Moreover, according to the WHO health report of the global population, 75% of them are claimed as subhealth and over 68.5% of these people are the health care workers during the COVID-19 pandemic.^80^ The demand for POC diagnostic platforms continues to increase. It is crucial to detect and monitor the biological and chemical molecules in tears under minimal concentrations within the physiological conditions through rapid and accurate methodologies in the modern world.^81^ Fluorescence biosensors, therefore, are among the target examples that can rapidly detect analytes. Reversibility of the fluorescence biosensors is also an important criterion for POC settings, as the patients require multisensing and real-time monitoring for ocular disease diagnosis. The evolution of typical fluorescence sensing technologies can access the opportunities in real-time monitoring, diagnosing specific ocular diseases, and understanding the physiological conditions within the eye systems.^29^ Typical fluorescence sensing techniques, such as Forster or fluorescence resonance energy transfer (FRET), can also apply one detection system for multianalyte monitoring in tears. In this circumstance, the ocular disease that is evaluated by different biomarkers and the accuracy of diagnosis can be improved for a future clinical study.

This review aims to summarize the applied clinical and experimental fluorescence sensing technologies for ophthalmic diagnosis and real-time monitoring in recent decades. The importance of fluorescence sensing for tear diagnosis will be discussed. The development of portable smart readout devices for sensing at POC settings will also be included. Moreover, the expected aspect for fluorescence tear sensing is overwhelmed by the possibilities for drug delivery.

## Fluorescence Sensing Technologies

In the fluorescence mechanism, normally the excitation of one molecule from the ground state (S_0_) to a singlet state (S_2_) would occur by absorption (eq 1); then the relaxation occurs by emitting photon energy to a lower energy state (S_1_) (eq 2). The absorption can be treated quantitatively using the Beer–Lambert Law. The ending state (S_1_) does not have to be the ground state, and the remaining energy within the molecule may be emitted through further fluorescence processes or dissipated by nonradiative relaxation energy such as heat. Therefore, the fluorescence process is rotative; the same fluorophore can be excited and detected repeatedly as long as the fluorophore is not destroyed at the excited state. Various principles have been applied to define a fluorescence process, including quantum yield, lifetime, quenching, photobleaching, and energy transfer.
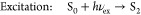
1
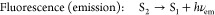
2where S_0_ indicates the ground state; S_1_ and S_2_ indicate singlet states; *h* indicates the Planck’s constant; ν_ex_ and ν_em_ indicate the frequency of the photon.

### Characteristics for Fluorescence

**Figure 2:**
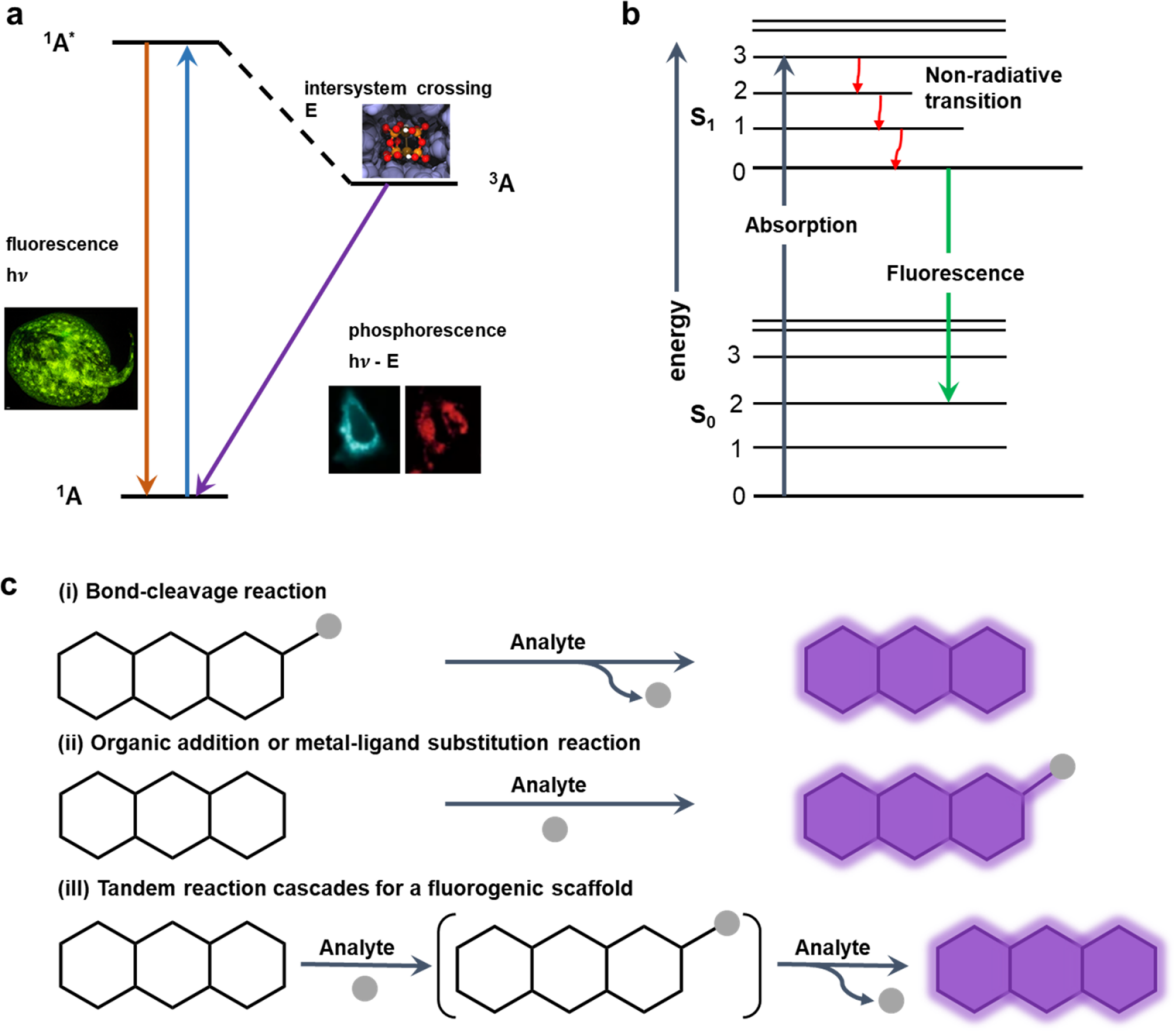
Schematic explanation for fluorescence and current fluorescent sensing methods. (a) The Jablonski diagram demonstrates one molecule (A) excited from the ground state (^1^A) followed by two procedures: including direct fluorescence and intersystem crossing to its triplet state (^3^A) phosphorescence to the ground state after that. The relative figures for different sections of the Jablonski diagram.^84−86^ Fluorescence image. Reproduced with permission from ref (84). Copyright 2014, Public Library of Science. Intersystem crossing image. Reproduced from ref (85). Copyright 2018, American Chemical Society. Phosphorescence image. Reproduced with permission from ref (86). Copyright 2020, Nature Portfolio. (b) Energy transfer for Jablonski diagram demonstration of fluorescence reaction. The system excitation reaction occurs electronically and vibrationally; then the high-energy photon is absorbed by an electron. The system relaxes by vibrational reactions, and the fluorescence at a longer wavelength is triggered eventually. (c) Relative common approaches for turn-on or radiometric fluorescence sensing detection mechanisms: (i) bond-cleavage reaction; (ii) organic addition or metal–ligand substitution reaction; and (iii) tandem reaction cascades for fluorogenic scaffolds.^87^

The fluorescence process usually can be visualized with a Jablonski diagram (Figure 2a) which illustrates molecular electronic states and demonstrates the transitions between them via a diagram. The states are arranged in two directions, the energy levels are measured vertically, and the spin multiplicity is grouped horizontally. Squiggly arrows indicate nonradiative transitions, and radiative transitions are noted by straight arrows (Figure 2b). The absorption spectrum and the emission image are mirror images for some fluorescent molecules and can be explained by the Frank–Condon principle.^82^ As a consequence, the vibrational levels are similar between the excited state and the ground state, and the nucleus does not move. Moreover, nonradiative transitions exhibit various mechanisms and with different labels in the diagram. The vibrational relaxation presents the relaxation from the molecule’s excited state to its lowest vibrational level. The isolated molecules do not exhibit this process, as the energy from the molecule to its surroundings would be dissipated. Furthermore, internal conversion (IC) and intersystem crossing (ISC) are also two types of nonradiative transition. An examination of the Jablonski diagram indicates that emission energy is universally less than absorption energy.^82^ As a consequence, lower energy level or longer wavelengths can motivate fluorescence reactions. Stokes shifts usually indicate the energy difference from the absorbed fluorescent molecule to the emitted fluorescent molecule. The rapid decay of vibration and the heat caused by extra vibrational energy are the initial causes for Stokes shifts (Figure 2).^83^ Additionally, excited state reactions, solvent effects, energy transfer, and complexation reactions can lead to further Stokes shifts.

The efficient rate of one fluorescence process is called the fluorescence quantum yield (QY) and is usually equal to the amount of emission photons divided by the amount of absorption photons.^79^ An alternative explanation of QY is by the decay rate of the excited state (eq 3), and the nonradiative rates that are caused by mechanisms are included where *k*_isc_ indicates ISC, *k*_ic_ indicates IC, *k*_pd_ and *k*_d_ indicates predissociation and dissociation respectively, and *k*_ec_ is the external coversion. Therefore, the fluorescence QY can be affected by the variation of the rate in any pathway. However, QY is independent or has less dependency on the wavelength of exciting radiation according to the Kasha–Vavilov rule, because the fluorescence emission usually takes place after the decay of excited molecule transfer to the lowest vibrational level.^82,88^ The excited state lifetime may also be influenced by the rate changes, and the fluorescence can be explained by a lifetime using first-order kinetics (eq 4). The fluorescence lifetime is critical for some applied fluorescent technologies, such as Förster resonance energy transfer (FRET) and fluorescence-lifetime imaging microscopy. A fluorophore’s fluorescence lifetime (τ) usually indicates an exponential decay of the radiative (*k*_f_) and nonradiative (*k*_nr_) process during the depopulation of excited state molecules (eq 5). Subsequently, with an infinitesimally short excitation process, the fluorescence intensity decay (*I*_t_) with time can be expressed (eq 6). The fluorochrome may experience different reactions, for instance, diffusion of molecules, reaction process of molecule through conformational changes, or molecular interactions with surrounding molecules during the lifetime. Consequently, it provides chances for lifetime measurements to probe these actions. Fluorescent lifetime is an important parameter for many tear sensing studies.^89,90^
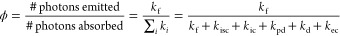
3
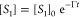
4
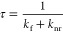
5
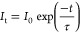
6where *k*_f_ indicates the rate constant of radiation with spontaneous emission; ∑_*i*_*k*_*i*_ indicates all rates of excited state decay in total; [S_1_] indicates excited state molecules’ concentrations at time *t*; [S_1_]_0_ indicates the original concentration and Γ indicates the decay rate; τ indicates the lifetime; *k*_nr_ indicates the rate constant of nonradiative decay process; *I*_0_ indicates the initial intensity, *I*_*t*_ indicates the fluorescence intensity at time *t*, and *t* indicates time.

Additionally, the fluorescence polarization can be measured through the orientation of the transition moment of fluorochromes at rapid emission, as the measurement of anisotropy can determine the rotation of fluorochromes. Fluorescence quenching, a phenomenon in which a molecule (quencher) interacts with the fluorophore, leads to a reduction in quantum yield or lifetime. Furthermore, autofluorescence is also one of the species in fluorescent sensing technology, and it occurs from cellular components with fluorescence properties instead of being developed from the fluorochrome of interest. The most typical example would be flavins and extracellular matrix components such as elastin, lipofuscin, and collagen.^91,92^ Photobleaching is an important photochemical process that can be applied in several practical fluorescence sensing technologies. The chemical reactivity of fluorochrome is high, and the fluorochrome lives longer than singlet states under its dark triplet excited state; hence, the photochemical process occurs predominantly.

Fluorescence can be characterized by different parameters and applied in various scientific areas. Fluorophores for fluorescent detection are important elements, as they obtain high specificity and satisfy basic principles. Fluorophores can be applied as diverse characters, which can be utilized alone as a substrate of enzymes, probes, or indicators. In the meantime, they can be bonded covalently to a macromolecule as a marker for bioactive reagents.^93−95^ Hence, various fluorescence technologies and their practical applications on the platform of tear fluids can be utilized for future personalized treatment and applied at POC settings.

### Fluorescence Sensors for Ophthalmic Diagnosis

#### pH and Electrolyte Sensing

**Figure 3:**
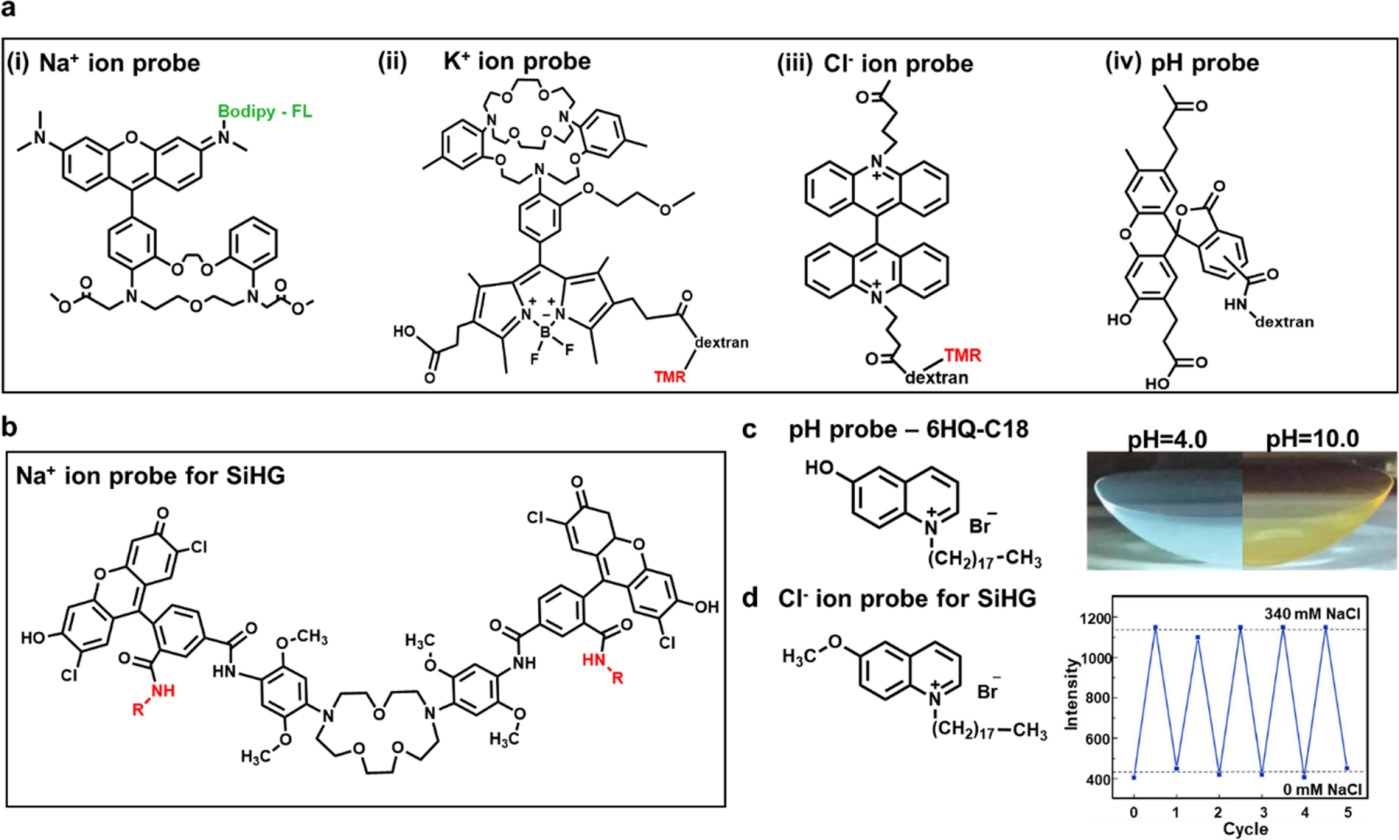
Chemical structures of the sensing probes for pH, sodium, potassium, and chloride ions and experimental results. (a) Chemical structure demonstration of fluorescent probes (i) Na^+^; (ii) K^+^; (iii) Cl^+^; and (iv) pH sensing probe. (b) Chemical structure of sodium ion detection within silicone hydrogel contact lens (SiHG). (c) pH sensing probe that was used for tear diagnosis (left) and the resulting fluorescent contact lens sensors (right); the blue color fluorescent contact lens was at pH = 4.0, and the yellow fluorescence image was obtained under pH = 10.0. Reproduced with permission from ref (89). Copyright 2017, Elsevier. (d) Reversibility test of the detection of Na^+^ and Cl^–^ ions. Reproduced with permission from ref (90). Copyright 2020, Elsevier.

Electrolytes and pH levels in tears have always been crucial and popular for tear sensing and diagnosis. The composition of the tear film is based on the ion and water transported from the ocular surface epithelia and secreted fluid from lacrimal glands. Hence, major electrolytes within tears (pH, Na^+^, K^+^, Ca^2+^, Mg^2+^, Cl^–^, and Zn^2+^) are the most popular analytes for real-time fluorescence monitoring in ophthalmic platforms. Moreover, variation of these electrolytes would be related with a series of ocular diseases, such as dry eye disease (DED), ocular infections caused by parasites, and thyroid eye disease.^96−98^ Besides the quantification of different ions for various ocular disease demonstrations, DED is one of the most common examples.^52,53^ Existing clinical diagnostic approaches for DED can be varied by symptom identification,^99^ ocular surface examination with aid of slit lamp,^100^ quantification tests (e.g., Schrimer’s test),^101−103^ physical tear fluid analysis (e.g., TearLab),^104^ and lateral flow assay detections (e.g., InflammaDry and Tearscan).^105,106^ However, most of the detection methods lack specificity, commercial availability, and understanding of pathophysiology of DED. The potential of tear fluid for DED diagnosis and differentiation should be explored. The development of fluorescent biosensing technologies would enable high selectivity for continuous detection of typical analytes and major ions within tears as well as achieving a relatively rapid and cheap detection. One fluorescence detection method was developed to analyze the concentrations of electrolytes in tear fluid from anesthetized mice.^107^ The dual-wavelength fluorescent indicators were selected accompanying the application of the ratio imaging fluorescence microscope. Both red- and green-colored fluorescence images were obtained from the self-designed dual fluorescent membrane-impermanent indicators for Na^+^ (Figure 3a(i)), K^+^ (Figure 3a(ii)), and Cl^–^ (Figure 3a(iii)), and a bis(carboxyethyl)-carboxyfluorescein fluorescence-conjugated dextran was utilized for pH detection (Figure 3a(iv)).^107^ The remarkable universal concentrations of electrolytes within the wild-type mice were reported, and the Na^+^ ion level was claimed to be significantly higher in AQP5 null mice and declined after the ocular surface was exposed to a humid environment.^107^ The *in vivo* fluorescence analytic method for tear analyte determination was conducted using the ratio fluorescence microscopic technology. The overall experiment provided the idea for specifically synthesized fluorophores applying for tear sensing and indicated the biocompatibility for *in vivo* measurement. However, the experimental element was anesthetized during the staining process, noninvasive detection could be considered and developed for commercial applications.

Instead of fluorescence sensing with the aid of injection through ocular surfaces, other types of devices have been studied and investigated during the recent decade. Wearable sensing technologies are therefore emerging and developed during recent years. One silicone hydrogel (SiHG) contact lens sensor was fabricated and examined to distinguish the concentration levels of pH, Na^+^, and Cl^–^ ion for ocular disease diagnosis.^89^ During the fabrication and evaluation procedure at the beginning stage of the SiHG sensor, the stabilities and other properties were examined for three sensing probes. As for the pH sensing probe, three chemicals including polarity-sensitive probes, 1-anilinonaphthalene-8-sulfonic acid (1,8-ANS, ANS), and a 4-(1-octylamine)-7-nitrobenzoxadiazole (NBD-C18) were used. Both hydrophilic and hydrophobic pH sensing probes were detected and compared to exhibit the sensing property of the fabricated probe. The chloride ion sensing was fabricated from 6-A methoxyquinolinium-containing (SPQ) probe.^89^ As for the mechanisms of pH and chloride ion sensing, hydrophobic and hydrophilic fluorescence interactions were detected and evaluated through the C18 and C3 alky chains. Among different types of contact lenses, SiHG contact lenses were selected for their high Dk value (permeability of the material) and silicone content (52%).^89^ The hydrophobic fluorophores for ion sensing were hardly removed from aqueous solutions due to a strong binding between SiHG lenses and the selected fluorophore. With a similar fabrication process, another tear sensing experiment was employed for sodium and chloride ions detection by SiHG lenses. The binding site of the Cl^–^-sensitive fluorophore between the octadecyl side chain and SiHG lenses was hydrophobic.^90^ As for the sodium-sensitive fluorophore, the sodium green (SG) and poly(l-lysine) (PL) were combined through covalent conjugation (Figure 3b). The fabricated contact lens sensor achieved a wider concentration range of detection for Na^+^ and Cl^–^ ions, and the response of each fluorescent was independent.

Moreover, a pH-independent SiHG was also detected with the selected labeled fluorophore and showed a significant fluorescence change under pH = 4 (blue) and pH = 10 (yellow) (Figure 3d). The reversibility of the fluorescence sensor was examined for Na^+^ and Cl^–^ ion detection as well (Figure 3d).^90^ By testing the fabricated fluorescent probe, 5 cycles were obtained and the contact lenses were rinsed various times within 3 mL of buffered solution in each cycle. The fluorescent lifetime was also detected between 0 and 340 mmol L^–1^ of NaCl and claimed no effect on different concentration of NaCl, where 0 mmol L^–1^ of NaCl indicated 1.5 ns and 340 mmol L^–1^ indicated 2.8 ns at fluorescence-reversible lifetime detection. The interference of tear proteins for the reversibility of the probe was also evaluated and reported no effect even after rinsing the lenses for 2 h within the tear proteins buffered solutions. The interfacial region of SiHG lenses were well-established and explored through these studies, and the developed silicone hydrogel fluorescent contact lens sensor can be established and integrated with other tear analytes for typical ocular disease diagnosis and monitoring of ophthalmic physiologies. Furthermore, the approved detection of reversibility of fluorophores for continuous tear monitoring enhanced the advantages of fluorescence tear monitoring by comparing with other tear diagnostic methods, such as disposable colorimetric test strips.

**Figure 4:**
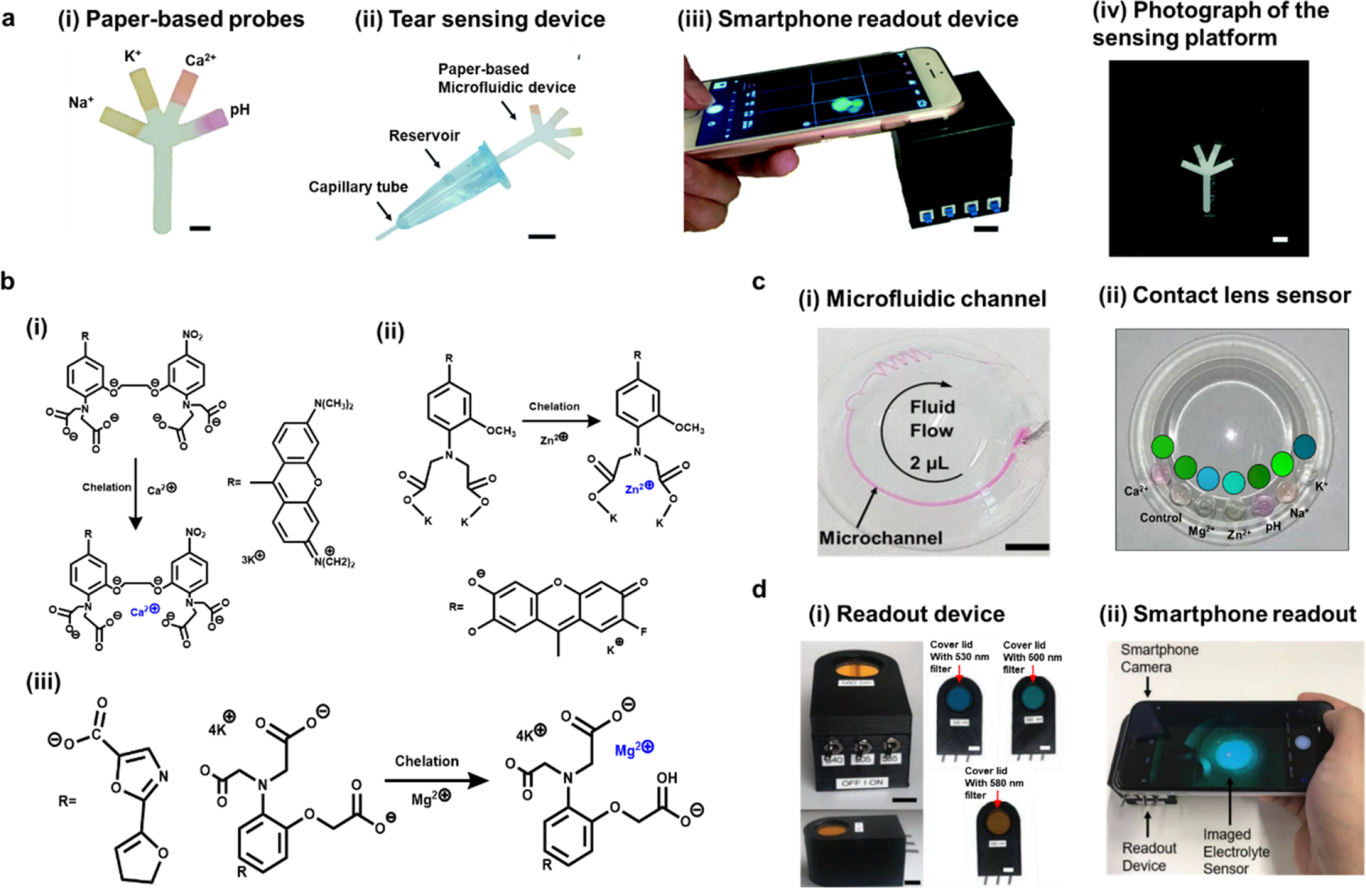
Multifluorescence sensing devices and fluorescence chelation reaction. (a) Paper-based microfluidic device for multianalyte sensing in tears: (i) fabricated paper-based sensing probes for pH, Na^+^, K^+^, and Ca^2+^ ion detection. (scale bar: 2 mm); (ii) finalized sensing device for tear diagnosis (scale bar: 1 cm); (iii) guidance of the smartphone readout device; scale bar = 1 cm; (iv) photograph for reading the paper-based sensing device within the black box before excitation of the fluorophores (scale bar: 4 mm). Reproduced with permission from ref (53). Copyright 2017, The Royal Society of Chemistry. (b) Chelation fluorescent reaction mechanisms for (i) Ca^2+^, (ii) Zn^2+^, and (iii) Mg^2+^. (c) Fabrication of microfluidic contact lens sensor: (i) closed microfluidic channel for 2 μL of fluid flowing in the microchannel; (ii) fabricated contact lens sensor for multianalytes detection. (d) Photograph of readout system: (i) readout box for multidetection of fluorescence sensors, consisting of three excitation switches of the filter and three readout emission filters with different wavelengths of light; (ii) smartphone readout demonstration for detection the ions within contact lens sensor. Reproduced with permission from ref (52). Copyright 2020, Wiley-VCH.

Quantitative analysis and real-time monitoring of analytes in tear fluid are essential for diagnosing eye diseases at the early stage for a POC platform, especially for tear ion detection. A type of paper-based fluorescence sensor for tear diagnosis was investigated with a multidetection mode strip for pH, Na^+^, K^+^, and Ca^2+^ ions using chelation reactions (Figure 4a(i,ii)).^53^ The fluorescence sensor was fabricated incorporating an optical readout device and a smartphone diagnostic system (Figure 4a(iii,iv)). As a continuous study for this type of sensor, one fluorescent scleral contact lens sensor was then fabricated to detect pH, Na^+^, K^+^, Ca^2+^, Mg^2+^, and Zn^2+^ ions within the tear physiological range. A smartphone readout was accompanied by this sensor for analyzing specific ion-sensitive data quantitatively.^52^ The constructed sensor is advantageous in the ability to recognize the severity of DEDs and their different types (Table 1). Chelation reactions were utilized for different fluorophore detection of individual concentrations (Figure 4b(i–iii)). The pH probe, for instance, was examined and evaluated in the concavity of the contact lens. Moreover, the Na^+^ sensing probe (15.6 mmol L^–1^) was selected by the crown ether derivatives within 0–100 mmol L^–1^ of the detection range, and the K^+^ sensing probe could be examined from 0 to 50 mmol L^–1^ with a LOD at 8.1 mmol L^–1^.^52^ Acid-based probes were utilized for Ca^2+^ and Zn^2+^ monitoring ranging from 0.50 to 1.25 mmol L^–1^ and from 0.5 to 0.8 mmol L^–1^ respectively. In addition, a sensitivity of 1 μmol L^–1^ was claimed for the Zn^2+^ ion sensor and the range of detection was 10–20 μmol L^–1^.^52^ Moreover, the microfluidic channel of the contact lens was examined for the final fabricated contact lens sensor (Figure 4c).

**Table 1:** Ion Detection within Tear for the Diagnosis of Dry Eye Disease and Its Subtypes^52,53^

		Abnormal Level within Tears (mmol L^–1^)			
		Dry Eye Disease Type			
Electrolytes	Detection Range within Tears (mmol L^–1^)	MGD	LGD	MGD and LGD	Sensitivity (mmol L^–1^)
pH	∼7.4	∼7.9			0.12
Na^+^	120–165	133.2–136.1	133.2–142.2	133.2–145.1	15.6
K^+^	20–42 (ave. 24)	24.6	24.9	25.4	0.8
Ca^2+^	0.4–1.1 (ave. 0.8)	0.82	0.84	0.86	0.02–0.05
Mg^2+^	0.5–0.9 (ave. 0.61)	0.61	0.63	0.65	0.01–0.03

The development of a portable readout device for data collection and process is another advantage of this series of research. This device was constructed with light-emitting diodes (LEDs) and bandpass optical filters for the sensor excitation. Data collection was another innovative point for the research (Figure 4d(i)). One smartphone camera was applied to assist with the fabricated readout device to deliver measurements quantitatively (Figure 4d(ii)). Then, the finalized fluorescent biosensor based on scleral lenses was explored for diagnosing and detecting the severity stage and distinguishing the subtypes of DEDs, meibomian gland dysfunction (MGD), and lacrimal gland dysfunction (LGD), for instance. The conversion of fluorescence into readable output data using a smartphone is advantageous in a personalized POC platform by providing the possibility for patients to collect data corresponding with their ocular conditions at any moment necessitated. Future development of the build-up application of smartphones can also be enriched with different sections of a variety of ocular diseases, and it would be user-friendly for real-time POC monitoring. Ascorbic acid, as an important antioxidant biomarker for ocular inflammations, has also been studied for fluorescence detections.^108,109^ However, the detection of ascorbic acid at POC platforms was conducted in aqueous humor instead of tear fluid with a high accuracy over than 80%.^108^ The sensor was mainly developed for diagnostics of ocular globe injuries and glaucoma care. Further fluorescence detection of ascorbic acid should be developed for tear fluid examination.

#### Glucose Sensing

**Figure 5:**
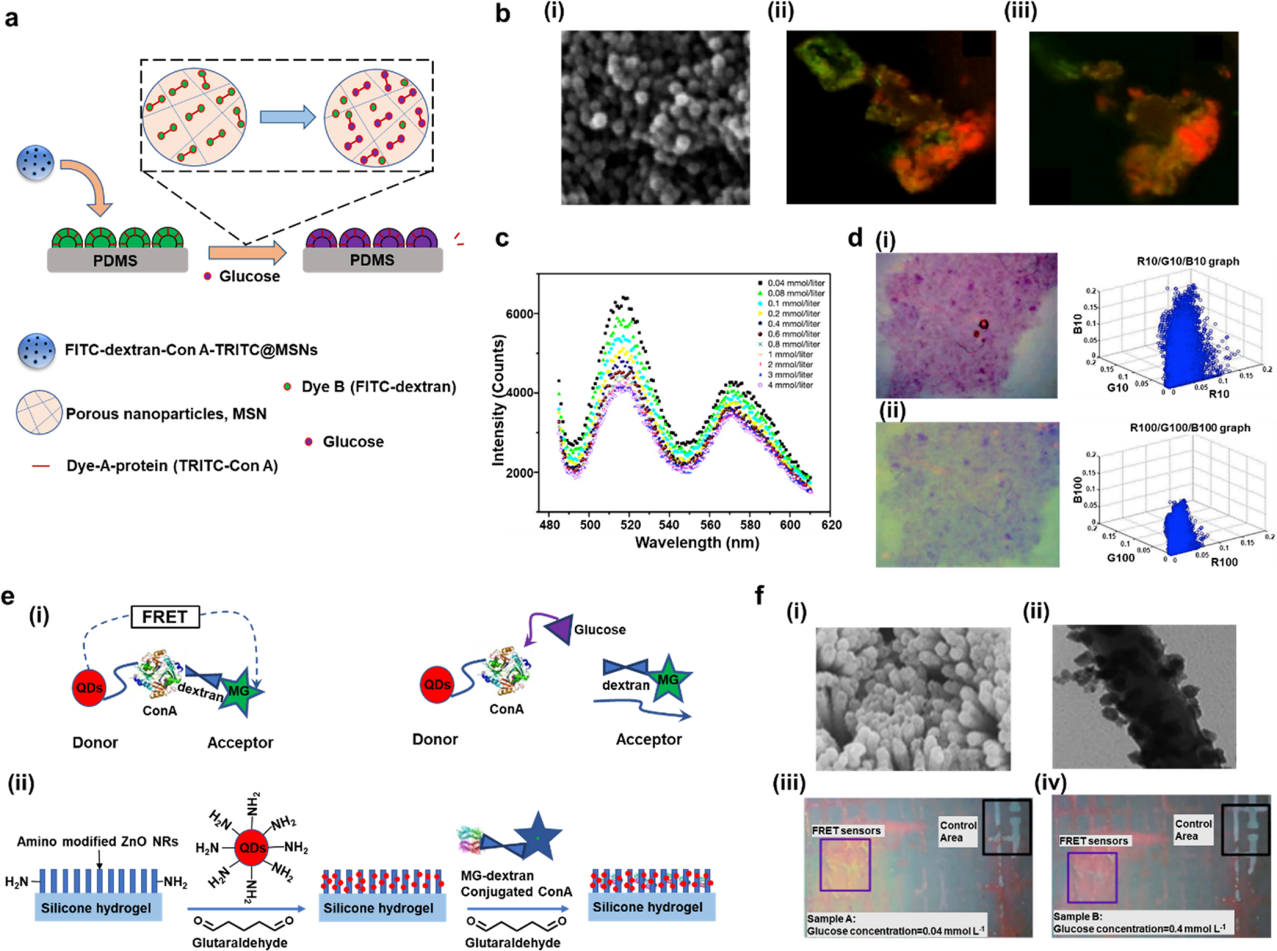
Highly sensitive glucose sensor based on FRET within tears. (a) Detection mechanism for tear glucose sensing within MSNs. (b) Microphotographs for the nanoparticles and aqueous glucose sensing: (i) micrograph (SEM) of assembled FITC-dextran-ConA-FRITX@MSNs; LCSM photograph of fabricated FRET sensor before (ii) and after (iii) the addition of glucose (20 μL) within 0.10 mmol L^–1^ of aqueous solution. (c) Fluorescent spectrum with various glucose concentrations. (d) Image of the FRET sensor and its conversion of readable MATLAB data under the glucose concentration of (i) 0.05 and (ii) 0.10 mmol L^–1^, respectively. Reproduced with permission from ref (127). Copyright 2013, SAGE. (e) Mechanism for tear glucose sensing: (i) illustrated FRET glucose detection included ConA conjugation quantum dots as the donor and MG as the acceptor; (ii) schematic mechanism of sensor immobilization on silicone hydrogel. (f) Images for fabricated nanoparticles and glucose sensing: (i) micrograph of QDs coated with ZnO nanorods using SEM; (ii) TEM image of QDs coated with ZnO nanorods; the resulting fluorescence images for the patterned FRET sensors on silicone hydrogel under aqueous glucose at 0.04 mmol L^–1^ (iii), and 0.4 mmol L^–1^ (iv). Reproduced from ref (128). Copyright 2017, Elsevier.

Glucose has been considered one of the critical biomarkers for diabetes diagnosis. There have been extensive studies conducted for glucose monitoring within various biological fluids, including blood,^110,111^ interstitial fluid,^112,113^ urine,^110,114,115^ sweat,^116−118^ saliva,^114,119^ and tear fluid in the recent decade.^120−124^ Tear glucose monitoring has been recently established for diagnosing diabetes and diabetic retinopathy. A normal level of tear glucose at 0.16 ± 0.03 mmol L^–1^ was claimed as compared to the diabetic patients 0.35 ± 0.04 mmol L^–1^, and the glucose level within tears is 0–5 mmol L^–1^.^125,126^ Different technologies have been applied for tear glucose sensing, especially fluorescence sensing technologies. Fluorescence resonance energy transfer (FRET) is one of the popular methods for low volume glucose detection. One highly sensitive nanostructured fluorescent biosensor was fabricated by utilizing FRET to monitor tear glucose levels (Figure 5a).^127^ In this work, the interaction between the selected nanoparticles FITC-dextran-silica and tetramethyl rhodamine isothiocyanate-labeled Concanavalin A (TRITC-Con A) occurred through binding Con A to dextran molecules. The FRET pairs were then formed by fluorescein isothiocyanate and tetramethyl rhodamine isothiocyanate (FITC-TRITC). The fluorescent chip device for tear glucose sensing was then formatted by the deposition of these fabricated fluorescent nanomaterials on the poly(dimethylsiloxane) (PDMS) surface. The insertion of glucose could replace the TRITC-Con A from PDMS. The different levels of glucose concentration would be detected through the fluorescence resonance energy transfer (FITC-TRITC) ratio. The morphology of assembled FITC-dextran-Con A-TRITC mesoporous silica nanoparticles (MSN) on PDMS was characterized using a scanning electron microscope (SEM) (Figure 5b(i)), and the diameter of the nanoparticles were indicated as 60 ± 5 nm on average. Further fluorescence images of FRET sensors from laser confocal scanning microscopy (LCSM) were also shown to demonstrate the glucose concentration (Figure 5b(ii,iii)). As a result, the finalized FRET biosensor could reach a detection range within 0.04–4 mmol L^–1^, and the data of the sensor could be obtained within 2 min (Figure 5c). Moreover, the chip can still be functionalized within 5 days.^127^ The obtained fluorescence image was finally converted into readable data with the aid of MATLAB coding. Different concentrations of glucose were evaluated, and the image and data of glucose under 0.05 mmol L^–1^ in Figure 5d(i) and 0.10 mmol L^–1^ in Figure 5d(ii) were the corresponding results, respectively. The binding performance of the fabricated sensor was further investigated within the hydrogel contact lens material; the fluorescence property and biocompatibility were proven. With the readable developed MATLAB data, it could be applied to smart readout devices for the future perspective.

Another similar fluorescence tear glucose sensor has been further developed by using fluorescent patterned arrays. The illustrated mechanism (Figure 5e(i)) introduced a FRET pair consisting of Con-A-conjugation quantum dots as a donor and MG as an acceptor, and the quenched fluorescence was restored by a competitive affinity of glucose over MG. The nanostructured FRET quenching sensor was immobilized onto ZnO nanorod arrays which were attached to the silicon hydrogel (Figure 5e(ii)).^128^ Both the fluorescence camera and the fluorometer could be utilized for analyzing glucose concentrations based on the uniquely designed procedure. During this fabrication procedure, the patterned ZnO nanorod arrays on the hydrogel were treated as a substrate, and ConA was conjugated onto the hybrid nanorods after conjugating CdSe/ZnS quantum dots (QDs) with ZnO nanorods. The fluorescence quenching molecule was claimed to be malachite green modified dextran and was bound onto Con A. After introducing the glucose, the dextran molecule was replaced by glucose competitively and the QDs fluorescence recovered. The QDs coated ZnO nanorod arrays were further analyzed by SEM (Figure 5f(i)) and transmission electron microscope (TEM) (Figure 5f(ii)). The FRET sensor on SiHG was evaluated with different concentrations of glucose (Figure 5f(iii–iv)), and a detection range of glucose was stated within 0.03–3 mmol L^–1^. The possibility of conducting a FRET sensor onto an SiHG was claimed. Hence, it would be promising to develop this series of FRET technologies onto an SiHG biosensor attached with contact lenses for real-time tear glucose monitor at POC settings. However, the biocompatibility for applying QDs to *in vivo* studies is needed to be considered.

**Figure 6:**
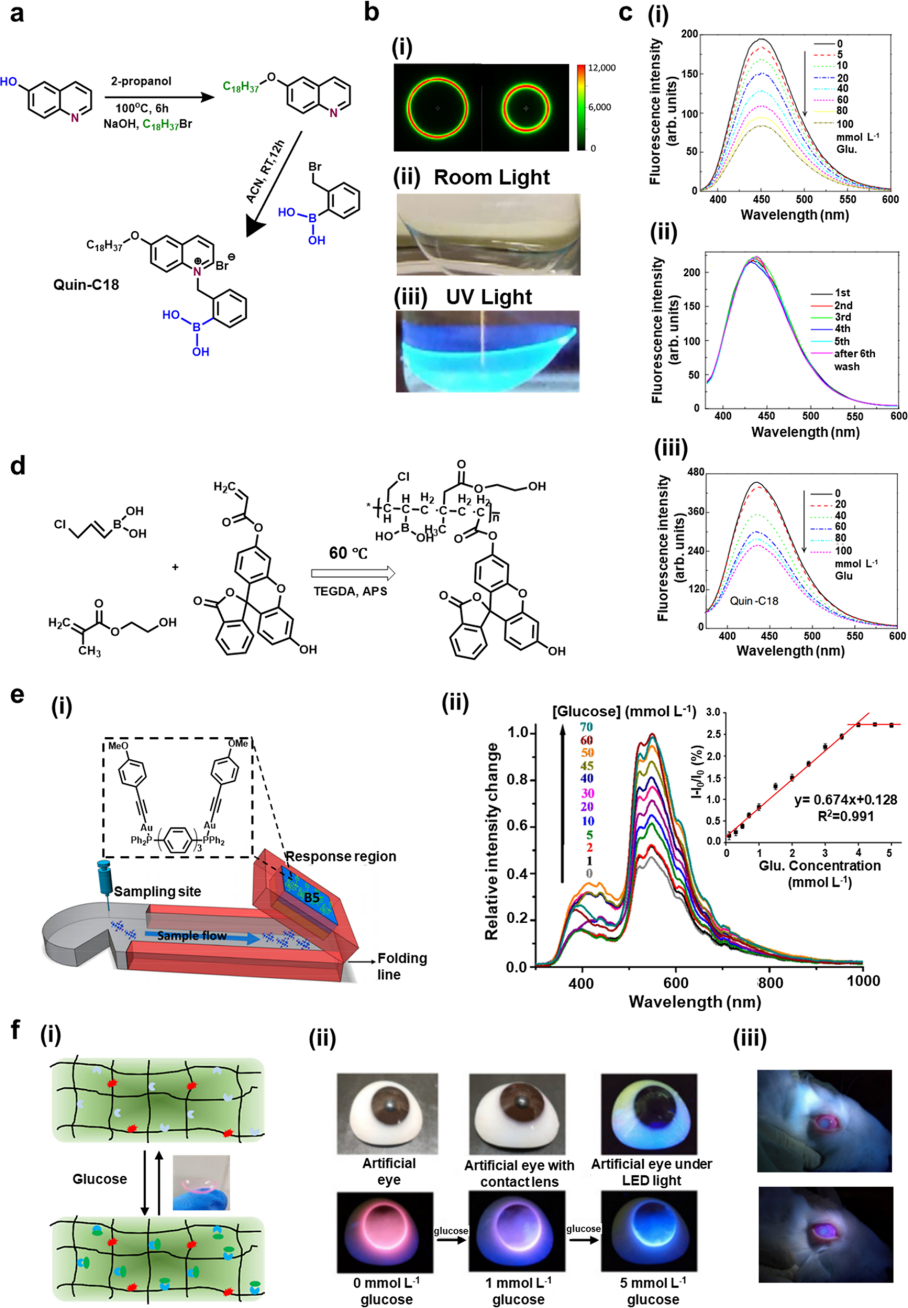
Fluorescence sensing of glucose in tears. (a) Synthetic mechanism for Quin-C18, the fluorophore that was used for glucose sensing. (b) Photographs for characterization of the contact lens glucose sensor: (i) micrograph for indicating the influence of dwell time over the increased intensity and phase angle, and the absence of the signal at the outside surrounding circles claimed the fluorophore could not be detected; photographs of Quin-C18-doped contact lens (Comfilcon A) under room light (ii) and UV light (iii) without an emission filter. (c) Glucose detection for optimization of contact lens sensor: (i) glucose concentration change versus the emission intensity in the phosphate-buffered solution (pH = 7.2); (ii) emission spectra for Quin-C18-coated Comfilcon A contact lens with multiple washing steps; (iii) finalized glucose-dependent emission results of Quin-18-coated Comfilcon A SiHG contact lens. Reproduced with permission from ref (129). Copyright 2018, Society of Photo-Optical Instrumentation Engineers Digital Library. (d) Mechanism for fluorescent copolymer formation for tear glucose sensing. (e) Paper-based microfluidic system for artificial tear glucose detection: (i) illustrated structure of a Schirmer test for tear glucose sensing; (ii) resulting emission spectrum for the variation of glucose detection under gel-encapsulated B5 sensing system; the inset chart indicated the calibration curve of glucose sensing within artificial tears (*n* = 3); here, the percentage emission change was evaluated with different glucose concentrations. Reproduced with permission from ref (130). Copyright 2018, Multidisciplinary Digital Publishing Institute. (f) Glucose-sensitive fluorescent contact lens sensor: (i) reversible detection mechanism on contact lens; (ii) photos for artificial eyes with contact lens and the fluorescent signal change with the variation of glucose; (iii) fluorescent image captured by smartphone from the rabbit with 1 mmol L^–1^ (upper) and 5 mmol L^–1^ (bottom) of glucose injection. Reproduced with permission from ref (131). Copyright 2022, Elsevier.

Instead of FRET imaging for glucose detection within tears, other contact lens sensors have been developed. For example, a typical SiHG contact lens sensor has been examined and fabricated for tear glucose monitoring continuously in the last five years. In this study, the monitoring mechanism was simplified, and a glucose-sensitive fluorophore (Glu-SFs) named Quin-C18 was utilized for examination.^126,129^ The interpenetrating polymer network of the SiHG lenses was evaluated for the characterization of the sensor during fabrication. One polarity-sensitive probe (Prodan) was applied for the lenses used within water and pure silicone regions. In order to confine the glucose-sensitive fluorescent probe within the interfacial area of the contact lens, Quin-C18 was formed and attached with one hydrophobic chain (Figure 6a).^129^ The applied glucose sensor would also suitable for *in vitro* examination and can be verified with different glucose concentrations (Figure 6b,c). The fabricated contact lens sensor was claimed to be consistent for glucose detection and proved that Quin-C18 can bound strongly to the lenses. Other properties such as leaching rate was also examined during detection. The leaching rate was extremely low after several rinsing processes, and a continuous detection result of glucose sensing was reported as being similar after a three-month storage of lenses within water. Hence, the developed glucose-sensitive sensor is advantageous in continuous POC detecting glucose within tears.

Therefore, the fabricated fluorescent contact lens sensor enables people to obtain the ophthalmic conditions and properties, and to explore the typical ophthalmological pathology through the examined results of specific tear biomarkers. Corresponding with the data-processing portable smart readout devices, it would be a solid benefit for clinical applications, personal diagnosis, and treatments for ophthalmic diseases. Another tear glucose fluorescent microfluidic paper-based analytical device was also developed based on lateral flow assay detection (Figure 6d,e), the fluorescent copolymer formation mechanism was developed and applied for the tear glucose sensing process. As a result, a detection range for the level of glucose in tears was achieved within the concentration between 0.1 and 4.0 mmol L^–1^.^130^ This sensor provided a faster response time and a wider range of glucose sensing. Meanwhile, it was claimed for practical diagnosis for diabetes. The development of the lateral flow assay detection provided an alternative measurement for glucose and diagnostic methodology to monitor the diabetic patients at POC platform.

One recent wearable glucose-sensitive fluorescent contact lens sensor was developed by integrating and immobilizing one glucose fluorescent probe and a reference fluorescent reference probe for calibration within the hydrogel network to achieve highly sensitive glucose detection on contact lens (Figure 6f).^131^ The fabricated glucose fluorescent contact lens sensor was able to recognize a concentration variation from 23 μmol L^–1^ to 1.0 mmol L^–1^ with a fluorescent color change from pink to blue (Figure 6f(ii).^131^ One smartphone RGB signal region was developed for collecting and transferring the fluorescent color of this experiment. Additionally, further *in vivo* rabbit experiments were conducted to indicate the biocompatibility of the fabricated contact lens sensor (Figure 6f(iii)). It is advantageous in providing a rapid and noninvasive method for real-time tear glucose examination, and the constructed glucose-sensitive contact lens sensor was able to detect as low as 9.3 μmol L^–1^ through the fluorescence spectrophotometer.^131^ The idea for implementing and stabilizing specific fluorescent probe for a target biomarker within the hydrogel network and attach it with a contact lens broadens the possibility for the detection of other tear analytes, especially for proteins within tear fluid. Most of the developed glucose fluorescent sensors can cover the detection range within the tear fluid and the achieved sensitivity of the sensor enable a differentiation ability between nondiabetic and diabetic individuals. However, few comparison experiments were conducted for *in vivo* identities before and after taking food. Because the tear glucose level is significantly lower than the glucose in blood (3.9–30 mmol L^–1^),^125^ it would be harder to achieve the variation examination within tears. Further developments could be made to reach a lower sensitivity of the fabricated fluorescent tear glucose sensor. Moreover, the clinical data collection and analysis for tears between healthy and diabetic groups of people would also benefit a future evaluation of the fluorescent tear glucose sensor.

#### Proteins Sensing

**Figure 7:**
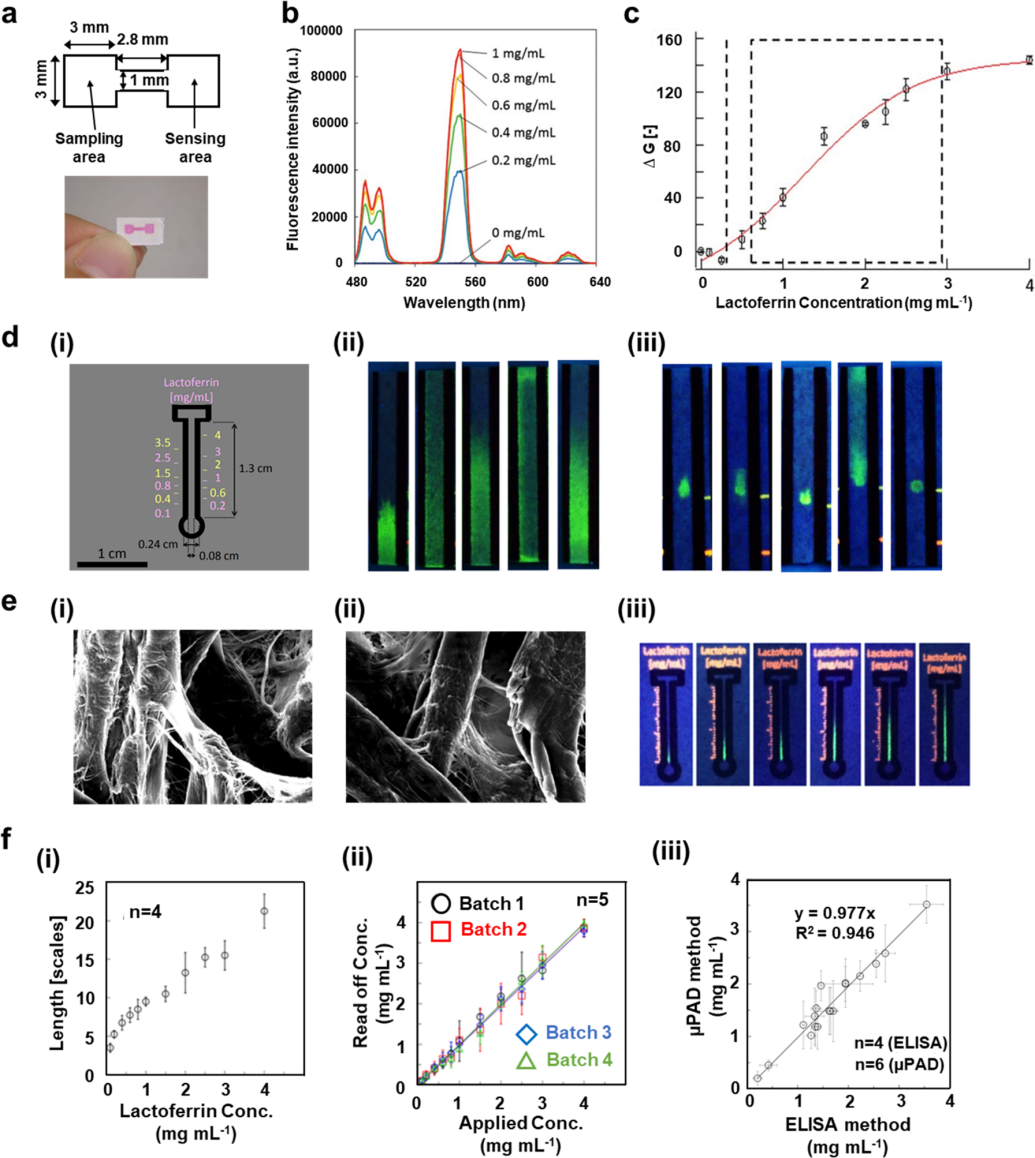
Developed fluorescent μPAD for tear LF detection. (a) Schematic (upper) and photograph (lower) of μPAD for tear LF analysis. (b) Emission spectrum of LF (0–1 mg mL^–1^) within a pH = 7.4 level of HEPES (50 mmol L^–1^) buffered solution. (c) Calibration curve of human LF on μPAD; the dashed line was LOD and the dashed square area represented regular the physiological detection range of the tear LF (*n* = 3). Reproduced with permission from ref (134). Copyright 2014, The Royal Society of Chemistry. (d) Distance μPAD for human LF detection: (i) illustrated outline structure of μPAD; (ii) UV illumination (λ_max_ = 254 nm) photographs of filter paper test for LF mobility within water; 100 mmol L^–1^ of NaCl aqueous solution; solution with lysozyme (3 mg mL^–1^); pseudotear fluid and water with treated lysozyme (3 mg mL^–1^), from left to right, respectively; (iii) UV illumination (λ_max_ = 254 nm) photographs for visualizing Tb^3+^-LF (1.5 mg mL^–1^, 0.5 μL) reaction after elution with different fluids: pure water, pseudotear fluid, LF in water and in the pseudotear fluid. Photograph on the right was the control results of LF buffered solution (HEPES 50 mmol L^–1^, pH = 7.4) without elution. (e) SEM of utilized filter paper (i) and results of the final fabricated μPAD; (ii) SEM for ι-Cg coated filter paper; (iii) images for analyzing different levels of LF (0.1, 0.6, 1, 2, 3, and 4 mg mL^–1^ from left to right) onto the ι-Cg coated μPAD. Scale bar: 10 μm. (f) Graphs for LF evaluations: (i) calibration curve between LF concentration and the length of emitted fluorescence line (0.5 mm was related to 1 scale of increment); (ii) further correlation on observed results for four sets of batches of fabricated μPAD; (iii) correlation curve between ELISA (*n* = 4) and μPAD (*n* = 6). Reproduced from ref (135). Copyright 2015, American Chemical Society.

Other than the integration of the fluorescent biosensor onto the contact lens, the microfluidic paper-based analytical device (μPAD) is also emerging for tear diagnosis. LF is one of the most important and abundant ion-binding proteins within the human body fluid, especially in tears.^132^ It is responsible for antibacterial and anti-inflammatory activities. Abnormal increase of LF levels within tears can lead to serious ocular diseases, for example, xerophthalmia and early-stage inflammatory bowel diseases.^132,133^ One fluorescent μPAD was developed for tear lactoferrin (LF) detection without antibodies (Figure 7).The inkjet printer and UV-curable ink were utilized for fabricating microfluidic patterns on μPADs.^55^ The fabricated fluorescent sensor exhibited a limit of detection (LOD) of LF at 0.3 mg mL^–1^ from 0.5 to 3 mg mL^–1^ of LF in the tear level.^134^ During the fabrication of μPAD, filter papers, A4 copy papers, and the EPSON inkjet printer were used. It was claimed to be important to attach the filter paper onto a sheet of copy paper, because the inkjet printer used for this work was not suitable to handle round shapes. The filter paper faces were then fitted with the round-shaped cut-out of the copy paper. During the inking process, both octadecyl acrylate and 1,10-decanediol diacrylate were applied for UV-curable ink. After the ejection of the paper from the printer, it was placed to cool at 10 °C.^134^ The filter paper with a circular cut area of 81 cm^2^ was utilized for microfluidic channel patterns. The designed sensor was then fabricated by a straight channel including two square areas (Figure 7a). During the detection, the sensing areas consisted of TbCl_3_ solution (1 mmol L^–1^) mixing with ethylene glycol (15 vol %); then the pattern was soaked in poly(vinyl alcohol) for 5 min.^134^ The paper was then dried to prevent LF adsorption on the paper surface. After that, the 25 mmol L^–1^ of the NaHCO_3_ solution was pipetted onto the sampling areas. The buffered solutions (HEPES pH 7.4, 50 mmol L^–1^) were used during all procedures. Finally, the single μPADs were cut from the papers after the soaking process of substrates. As a result, the LF was first detected within the buffered solution from 0 to 1 mg mL^–1^ (Figure 7b) and the paper-based device was examined with the obtained range of 0.63–2.9 mg mL^–1^ (Figure 7c).^134^ Instead of conducting the measurement with a signal readout instrument, distance-based LF was also developed for similar research (Figure 7d). Further treatment on the filter paper used was claimed (Figure 7e). With the fabricated ι-Cg (ι-carrageenan)-coated filter paper (Figure 7e(iii)), an even lower LOD at 0.1 mg mL^–1^ of LF was obtained.^135^ The LF within tears was eligible for detection with 0–4 mg mL^–1^ of LF with this μPAD (Figure 7f(i)). Instead of correlation from the fabricated device (Figure 7f(ii)), further evaluation was conducted between the standard ELISA test and μPADs (Figure 7f(iii)). After the success of detection in tears using μPADs, the device can be explored further by integrating paper-based sensors onto contact lenses or other portable devices to fulfill the aim at the POC diagnostic platform for ocular diseases.^26^ In addition, with the integration and investigation of fluorescent paper-based detection, more protein biomarkers within tear fluid including interleukin 6 (IL-6) and immunoglobin G (IgG) can obtain the potential for fluorescence detection with relative technologies, such as immunofluorescent assay and encapsulation of nanocluster fluorescence detection.^136,137^

#### Enzymes Sensing

**Figure 8:**
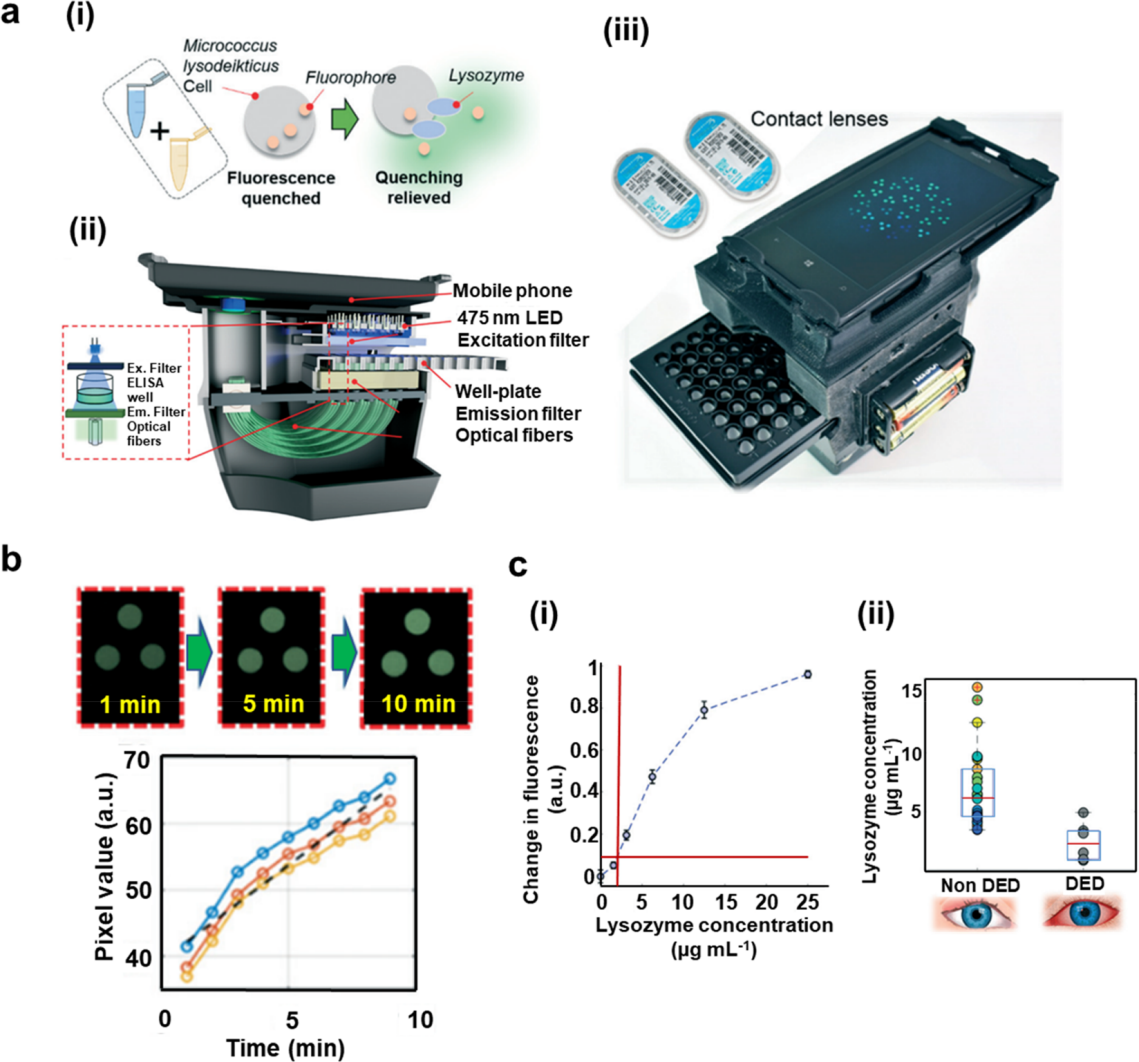
Detection of lysozyme within tears using developed fluorescence mobile phone-based microplate reader. (a) Detection method and schematic demonstration of well-plate reader developed by smartphone, (i) the final step of mixing the wash solution and the *Micrococcus lysodeikticus* cell solution within the ELISA well for fluorescent detection; (ii) a schematic illustration of the smartphone-based well-plate reader; (iii) photograph of the fabricated product. (b) The recorded results for fluorescent observation with time dependence, where the green channel indicated the fiber bundled image that was taken from the smartphone (three fibers per well) (upper) and the lower graph indicated the standard curve for fluorescence well-plate assay over time (10 min). (c) Experimental results for lysozyme detection: (i) Standard curve for fluorescence well-plate assay: the vertical and horizontal lines state the LOD (1.99 μg mL^–1^); (ii) overall resulting data of lysozyme concentration detection for healthy participants (*n* = 30) without DED for over 5-day monitoring period of contact lens wear and one-time measurement for patients (*n* = 6) with DED. The red horizontal line claimed the median concentration of lysozyme in each measurement group and the top and bottom line of the blue rectangular represented 75% and 25% of the data, respectively. Reproduced with permission from ref (138). Copyright 2020, The Royal Society of Chemistry.

Instead of proteins detected within the tear, enzymes have also been studied for fluorescence detection within tears. One contact lens sensing method was developed for sensing analytes within tears. The contact lenses were treated as collectors of samples, and the subsequent analysis was accompanied by one field-portable and cost-effective reader (Figure 8a(i)–(iii)).^138^ Lysozyme, as one of the most prevalent and important naturally occurring enzymes within tears,^139^ was therefore selected to be quantified. Moreover, the time-lapse imaging technology was utilized with the mobile reader to observe the increase of fluorescence signal within a standard well-plate. The obtained data was indirectly inferred to the change of lysozyme concentration through a standard curve. The best-suited contact lens and the assay were chosen empirically for tear collection and detection. The variation of lysozyme concentrations were then monitored within nine healthy human objectives over 2 weeks. The results were used for a comparison with the objectives with DED. A time dependency experiment was conducted with a mobile-based microplate readout that exhibited the data of each ELISA well with three fibers to get the green channels (Figure 8b). The fluorescence data increased over 10 min, and a calibration curve was delivered from the constructed smartphone reader with the variation of lysozyme concentration. The experimental data were then compared with the *in vivo* human objectives with and without DEDs (Figure 8c). As a result, the concentration of lysozyme increased from 6.89 ± 2.02 μg mL^–1^ to 10.72 ± 3.22 μg mL^–1^ (mean ± SD) was observed for six participants of nine who wear contact lenses regularly, and these objectives were detected with the induction of a digital ocular strain model during the period of contact lens wear. Moreover, a lower mean lysozyme level of a patient with DED was claimed compared to the healthy participants, the mean levels of concentrations were 2.43 ± 1.66 μg mL^–1^ and 6.89 ± 2.02 μg mL^–1^, respectively. The main advantages of this study would be introducing a simple and noninvasive sampling method for detection as well as the measurement system was considered to be relatively rapid, user-friendly, and cost-effective for indicating the physiological change within human ocular objectives. Future tear-fluid studies could be conducted with the application of this methodology, and it would be significant for tear biomarker multiplex measuring on a POC platform.

Another type of fluorescent detection of lysozyme within tears was developed through the inner filter effect of gold nanoparticles on CdTe quantum dots.^140^ This sensor was fabricated by utilizing lysozyme reacting with the lysozyme binding aptamer to avoid the reaction between gold nanoparticles and the lysozyme binding aptamer. The gold nanoparticles in this case would aggregate and a strong blue fluorescence could be observed under this circumstance. The finalized sensor was examined and evaluated within real tear and saliva samples, and the detection range was claimed between 1.0 nmol L^–1^ and 20 nmol L^–1^.^140^ The fabricated sensor is advantageous in high sensitivity and label-free detection of lysozyme. With the application of this method, further integration of this sensor onto paper-based microfluidic detection could be applied for POC detection. However, further biocompatibility tests should be qualified if the sensor could direct contact with human eyes.

## Future Prospective

During the past decade, ophthalmology-related fluorescence technologies have been commonly studied and have been explored for ophthalmic diagnosis from spectroscopic technologies to portable biosensors. The possibility of integration of smart readout devices and cooperation with a hand-held camera for fluorescence tear sensing has also been proposed that would be helpful for monitoring and diagnosis on POC platforms. Ophthalmic fluorescent monitoring has been well-established in lab-based studies (Table 2). Nevertheless, the detected analytes within tears are still limited in glucose and typical ions.^26,61,141,142^ More proteins and enzymes need to be explored for further ocular diagnosis, such as reactive oxygen species (ROS), immunoglobulins, and interleukins.^143^ Nanoscale carriers in fluorescent detection on targeted biomarkers in ophthalmologic diagnosis and drug delivery have received much attention recently, especially for relatively large-sized proteins and cytokines.^144−146^ Relative fluorescent technologies can also be employed for tear analyte detection, such as quantum dots, carbon dots, and FRET. As discussed, different means of tear sensing platforms are also one of the future directions for bringing biosensors to the commercial industry. For instance, paper-based detection, lateral flow assays (LFA), capillary tube detection, and three-dimensional (3D) printing could be established and combined with fluorescence sensing technologies to achieve personalized treatments on POC platforms.^147^ Biocompatibility should be one of the criteria for evaluation of the sensing platforms developments. Therefore, the development for biologically modified nanoparticles could also be one of the directions for ophthalmic therapeutic monitor and examinations, especially for diagnosing and monitoring cancer-derived ocular diseases.^148,149^

**Table 2:** Conclusion of the Developed Fluorescent Sensors for Tear Biomarker Detection

Tear Biomarker	Detection Platform	Sensitivity	Linearity	Animal Test	Response/Reaction Time	ref
pH	Silicone hydrogel contact lens	-	4.2–10.0	No	-	(89)
	Paper-based microfluidic channel	-	7.0–8.0	No	-	(53)
	Scleral contact lens	0.12	7.0–8.0	No	-	(52)
Na^+^	Silicone hydrogel contact lens	0.2–0.3 mmol L^–1^	0–150 mmol L^–1^	No	-	(90)
	Paper-based microfluidic channel	1.5 mmol L^–1^	0–200 mmol L^–1^	No	-	(53)
	Scleral contact lens	15.6 mmol L^–1^	0–100 mmol L^–1^	No	-	(52)
K^+^	Silicone hydrogel contact lens	-	0–200 mmol L^–1^	No	-	(90)
	Paper-based microfluidic channel	0.9 mmol L^–1^	0–50 mmol L^–1^	No	-	(53)
	Scleral contact lens	8.1 mmol L^–1^	0–50 mmol L^–1^	No	-	(52)
Ca^2+^	Paper-based microfluidic channel	0.03 mmol L^–1^	0–2 mmol L^–1^	No	-	(53)
	Scleral contact lens	0.02–0.05 mmol L^–1^	0.50–1.25 mmol L^–1^	No	-	(52)
Mg^2+^	Scleral contact lens	0.01–0.44 mmol L^–1^	0.5–0.8 mmol L^–1^	No	-	
Zn^2+^		0.001 mmol L^–1^	0.01–0.02 mmol L^–1^	No	-	
Glucose	Matlab imaging	-	0.04–4 mmol L^–1^	No	<2 min	(127)
	Fluorescence spectrum	-	0.03–3 mmol L^–1^	No	30 s	(128)
	Silicone hydrogel contact lens	-	0–100 mmol L^–1^	No	-	(129)
	Paper-based microfluidic channel	0.08 mmol L^–1^	0.1–4.0 mmol L^–1^	No	0.3 s	(130)
	Contact lens	0.0093 mmol L^–1^	0.023–1.0 mmol L^–1^	Yes	3–5 s	(131)
Lactoferrin	Paper-based microfluidic channel	0.3 mg mL^–1^	0.5–3 mg mL^–1^	Yes	15 min	(134)
		0.1 mg mL^–1^	0–4 mg mL^–1^	Yes	<10 min	(135)
Lysozyme	Contact lens	1.99 μg mL^–1^	0–25 μg mL^–1^	Yes	10 min	(138)
	Fluorescence spectrum	0.55 nmol L^–1^	1.0–20 nmol L^–1^	Yes	100 s	(140)

It was predicted that the global contact lens market will increase to over $19 billion in 2024.^150^ Instead of converting the contact lens for existing therapeutic and cosmetic uses, various countries have begun treating contact lenses as potential medical tools for broad applications in ocular-related disease diagnosis and drug delivery. Cooperation with a novel series of hand-held readout smart devices to enhance the facilities of minimally or noninvasive fluorescence contact lens sensors even brought a higher value for these novel generated contact lens sensors. The market for these kinds of smart contact lens sensors is claimed to possess over $24.12 billion by 2029.^150,151^ Therefore, both manufacturers and patients worldwide could obtain a promising potential market with the development of contact lens sensors. However, the low volume and concentration of sampled proteins and enzymes in tears are still negligible.^152,153^ Principles of fabricating fluorescence biosensors for in situ contact lens sensing to achieve POC in indication and real-time detection of ocular diseases should focus on the mechanism of sensing technologies, types of cross-linkers, and fluorophores. Another crucial ultimate prospective of fluorescence tear sensors is drug dosage and delivery. Microneedle arrays, as a popular developing technology in delivering drug, biosensing analytes, and neural interfaces,^154−156^ can be utilized to combine with fluorescence sensing technologies for drug delivery in tears. One of the advantages of the microneedle fabrication process is an easy operation by using low-cost 3D printing techniques.^157,158^ Subsequently, the fabricated fluorescent microneedle arrays can reach not only the tear fluid interface but other intraocular positions, such as aqueous humor. Then, one suitable design could be selecting each one-phase monitoring and diagnosis for ocular disease and combining the single test channel into the multidetection phase, simulating and optimizing the interferences for the final diverse sensing mode within the fluorescent sensor, and finally producing one microneedle channel for target therapeutic aim. Moreover, the experimental data can be compared and optimized with the obtained clinical ophthalmic technologies, such as ophthalmic spectroscopies and clinical chemistry analyzer, for instance. With the golden standard comparisons, the permitted tolerance of errors would be optimized to be as minor as possible.

## Conclusion

In conclusion, tear fluid detection has attracted continuous attention in scientific, technological, and clinical studies of healthcare diagnosis. Fluorescence sensing materials have been well-established for wide applications in the past decade. Fluorescent sensing in tear fluid offers a sensitive, cost-effective, and noninvasive platform for early diagnosis of ocular-related diseases, including various cancers, neurological disorders, sclerosis diseases, and Parkinson’s disease. One immediate future area could be directly working on immediate diagnosis and data collection for ocular disease at the POC platform in which the patients would not need to rely heavily on hospitality and could achieve self-detection and data collection. The development of biological assays would also be one of the intermediate future directions on ocular disease monitoring. The integration of the portable readout devices and wearable sensing tools (contact lens) can be helpful at the POC platform to improve the user experience and convenience. The fluorescence sensors then should be sufficient in sensitivity, selectivity, accuracy, and reproductivity. Hence, potential principles for fluorescence sensing and sufficient fluorophores need to be developed and investigated. Moreover, the biocompatibility of the fluorescent detection within tears should be considered for future commercialized trials. As for the intermediate future of fluorescent tear sensing, more complicate biomarkers within tears and even aqueous human fluid such as BDNF, IL-6, and other proteins need to be explored. Some neuro-related diseases could be achieved at a POC diagnosis and monitor. Moreover, a multichannel diagnosing fluorescent sensing could be developed to achieve multidetection on one ocular-related disease and that would lead to a more accurate data outcome for patients.

It is also necessary to achieve minimally or noninvasive tear sensing and real-time monitoring for ophthalmic diagnosis and ocular physiological index analysis. The achievement of drug delivery at the POC platform is one of the most critical longer future directions to tear diagnosis. Fluorescence contact lens sensors, as one of the most effective techniques for biosensing and real-time monitoring, therefore, should be used to conduct more investigations in intermediate ophthalmological diagnosis and long-term database collection for hospitalization. Sufficient fluorescence sensing technologies as well as the cooperation with portable hand-held smart devices should be evaluated and explored to enhance the functions of fluorescence sensing and diagnosis within tears at the POC platforms. These fabricated fluorescent sensors could be applied clinically for real-time monitoring, one-time detection, drug delivery, etc. In some cases, the continuous monitoring would not be necessary, but the annual or regular detection and monitoring would be required. For example, some glaucoma patients would need to do the examination every 6 months, where some of the ocular diseases such as inflammation would require a more frequent detection (once per week). Furthermore, real-time monitoring would be beneficial to the surgical monitoring before and afterward.

## Vocabulary

**Fluorescent sensing techniques**: Technologies that can be utilized for the detection of different analytes based on fluorescence mechanisms.
**Point-of-care (POC)**: Normally can be defined as medical applications and diagnostic methods for patients to use by their side at any time.
**Tear biomarkers**: Analytes in the tear fluid which can be examined and evaluated for ocular-related disease diagnostic and therapeutic treatments. The analytes include electrolytes, proteins, enzymes, and cytokines.
**Tear sensing and diagnostics**: This term is commonly applied to medical applications (ophthalmic research); the ocular-related disease can be diagnosed by the quantitative and qualitative analysis of tear biomarkers. Typical ocular diseases have been studied and evaluated, such as dry eye diseases, glaucoma, and diabetic retinopathy.
**Microfluidic paper-based sensing device**: The paper-based sensing device is a platform for medical, chemical, and biological analysis, and microfluidic channels are the media for achieving multidetection of analytes for the detection and differentiation of a wide range of ophthalmic diseases.
**Contact lens sensor**: The device or developed system that detects ophthalmic environments and physiologic variations and transfers the information through contact lens to the media by different methodologies, for example fluorescent contact lens sensors.

## References

[ref1] Lazar A. M.; Baritz M. I. Some Considerations on the Composite Structure of the Human Eye. Macromol. Symp. 2020, 389 (1), 1900103 10.1002/masy.201900103.

[ref2] Fricke T. R.; Tahhan N.; Resnikoff S.; Papas E.; Burnett A.; Ho S. M.; Naduvilath T.; Naidoo K. S. Global Prevalence of Presbyopia and Vision Impairment from Uncorrected Presbyopia: Systematic Review, Meta-analysis, and Modelling. Ophthalmology 2018, 125 (10), 1492–1499. 10.1016/j.ophtha.2018.04.013. 29753495

[ref3] Bourne R. R. A.; Flaxman S. R.; Braithwaite T.; Cicinelli M. V.; Das A.; Jonas J. B.; Keeffe J.; Kempen J. H.; Leasher J.; Limburg H.; Naidoo K.; Pesudovs K.; Resnikoff S.; Silvester A.; Stevens G. A.; Tahhan N.; Wong T. Y.; Taylor H. R.; Bourne R.; Ackland P.; Arditi A.; Barkana Y.; Bozkurt B.; Braithwaite T.; Bron A.; Budenz D.; Cai F.; Casson R.; Chakravarthy U.; Choi J.; Cicinelli M. V.; Congdon N.; Dana R.; Dandona R.; Dandona L.; Das A.; Dekaris I.; Del Monte M.; Deva J.; Dreer L.; Ellwein L.; Frazier M.; Frick K.; Friedman D.; Furtado J.; Gao H.; Gazzard G.; George R.; Gichuhi S.; Gonzalez V.; Hammond B.; Hartnett M. E.; He M.; Hejtmancik J.; Hirai F.; Huang J.; Ingram A.; Javitt J.; Jonas J.; Joslin C.; Keeffe J.; Kempen J.; Khairallah M.; Khanna R.; Kim J.; Lambrou G.; Lansingh V. C.; Lanzetta P.; Leasher J.; Lim J.; Limburg H.; Mansouri K.; Mathew A.; Morse A.; Munoz B.; Musch D.; Naidoo K.; Nangia V.; Palaiou M.; Parodi M. B.; Pena F. Y.; Pesudovs K.; Peto T.; Quigley H.; Raju M.; Ramulu P.; Resnikoff S.; Robin A.; Rossetti L.; Saaddine J.; Sandar M. Y. A.; Serle J.; Shen T.; Shetty R.; Sieving P.; Silva J. C.; Silvester A.; Sitorus R. S.; Stambolian D.; Stevens G.; Taylor H.; Tejedor J.; Tielsch J.; Tsilimbaris M.; van Meurs J.; Varma R.; Virgili G.; Volmink J.; Wang Y. X.; Wang N.-L.; West S.; Wiedemann P.; Wong T.; Wormald R.; Zheng Y. Magnitude, temporal trends, and projections of the global prevalence of blindness and distance and near vision impairment: a systematic review and meta-analysis. Lancet Glob. Health 2017, 5 (9), e888–e897. 10.1016/S2214-109X(17)30293-0. 28779882

[ref4] WHO World Report on Vison; World Health Organization, 2019; p 180.

[ref5] Gordois A.; Cutler H.; Pezzullo L.; Gordon K.; Cruess A.; Winyard S.; Hamilton W.; Chua K. An estimation of the worldwide economic and health burden of visual impairment. Glob. Public Health 2012, 7 (5), 465–481. 10.1080/17441692.2011.634815. 22136197

[ref6] Bielory L.; Syed B. A. Pharmacoeconomics of anterior ocular inflammatory disease. Curr. Opin Allergy Clin Immunol. 2013, 13 (5), 537–542. 10.1097/ACI.0b013e328364d843. 23945174

[ref7] Köberlein J.; Beifus K.; Schaffert C.; Finger R. P. The economic burden of visual impairment and blindness: a systematic review. BMJ. Open 2013, 3 (11), e003471–e003471. 10.1136/bmjopen-2013-003471. PMC3822298 24202057

[ref8] Cordero I. Understanding and looking after a retinoscope and trial lens set. Community Eye Health J. 2017, 30 (98), 40. PMC5646585 29070929

[ref9] Ye L. Y.; Jiang L. H.; Zhang L. H.; Karp L. C.; Zhong L. J.; Tao L. A.; Shao L. Y.; Wang L. J. Resolution of Slit-Lamp Microscopy Photography Using Various Cameras. EYE CONTACT LENS 2013, 39 (3), 205–213. 10.1097/ICL.0b013e318286bc0f. 23538733

[ref10] Bennett T. J.; Barry C. J. Ophthalmic imaging today: an ophthalmic photographer’s viewpoint – a review. Clin. Experiment. Ophthalmol. 2009, 37 (1), 2–13. 10.1111/j.1442-9071.2008.01812.x. 18947332

[ref11] Salmon J. F., 15 - Gonioscopy. In Glaucoma, 2nd ed., Shaarawy T. M.; Sherwood M. B.; Hitchings R. A.; Crowston J. G., Eds.; W.B. Saunders: 2015; pp 169–178.

[ref12] Schoenfeldt-Lecuona C.; Kregel T.; Schmidt A.; Pinkhardt E. H.; Lauda F.; Kassubek J.; Connemann B. J.; Freudenmann R. W.; Gahr M. From Imaging the Brain to Imaging the Retina: Optical Coherence Tomography (OCT) in Schizophrenia. Schizophr. Bull. 2016, 42 (1), 9–14. 26048298 10.1093/schbul/sbv073 PMC4681543

[ref13] Shirazi M. F.; Wijesinghe R. E.; Ravichandran N. K.; Kim P.; Jeon M.; Kim J. Dual-path handheld system for cornea and retina imaging using optical coherence tomography. Opt. Rev. 2017, 24 (2), 219–225. 10.1007/s10043-016-0288-5.

[ref14] Marschall S.; Sander B.; Mogensen M.; Jørgensen T. M.; Andersen P. E. Optical coherence tomography—current technology and applications in clinical and biomedical research. Anal. Bioanal. Chem. 2011, 400 (9), 2699–2720. 10.1007/s00216-011-5008-1. 21547430

[ref15] Mazlin V.; Xiao P.; Dalimier E. n.; Grieve K.; Irsch K.; Sahel J.-A.; Fink M.; Boccara A. C. In vivo high resolution human corneal imaging using full-field optical coherence tomography. Biomed. Opt. Express 2018, 9 (2), 557–568. 10.1364/BOE.9.000557. 29552393 PMC5854058

[ref16] Chandra S.; Rasheed R.; Sen P.; Menon D.; Sivaprasad S., Inter-rater reliability for diagnosis of geographic atrophy using spectral domain OCT in age-related macular degeneration. Eye, 2021. 10.1038/s41433-021-01490-5 PMC8807831 33686233

[ref17] Ehlers J. P.; Clark J.; Uchida A.; Figueiredo N.; Babiuch A.; Talcott K. E.; Lunasco L.; Le T. K.; Meng X.; Hu M.; Reese J.; Srivastava S. K. Longitudinal Higher-Order OCT Assessment of Quantitative Fluid Dynamics and the Total Retinal Fluid Index in Neovascular AMD. Transl. Vis. Sci. Technol. 2021, 10 (3), 29–29. 10.1167/tvst.10.3.29. PMC7995350 34003963

[ref18] Tao A.; Shao Y.; Zhong J.; Jiang H.; Shen M.; Wang J. Versatile optical coherence tomography for imaging the human eye. Biomed. Opt. Express 2013, 4 (7), 1031–1044. 10.1364/BOE.4.001031. 23847729 PMC3704085

[ref19] McCabe M. J.; Croce J. K. Optical Coherence Tomography. Circulation 2012, 126 (17), 2140–2143. 10.1161/CIRCULATIONAHA.112.117143. 23091086

[ref20] Choplin N. T.; Craven E. R.; Reus N. J.; Lemij H. G.; Barnebey H., 21 - Retinal Nerve Fiber Layer (RNFL) Photography and Computer Analysis. In Glaucoma, 2nd ed., Shaarawy T. M.; Sherwood M. B.; Hitchings R. A.; Crowston J. G., Eds.; W.B. Saunders: 2015; pp 244–260.

[ref21] Nghiem A. Z.; Nderitu P.; Lukic M.; Khatun M.; Largan R.; Kortuem K.; Balaskas K.; Sim D. Comparing diabetic retinopathy lesions in scanning laser ophthalmoscopy and colour fundus photography. Acta Ophthalmol. 2019, 97 (8), e1035–e1040. 10.1111/aos.14106. 31286663

[ref22] Zhang B.; Li N.; Kang J.; He Y.; Chen X.-M. Adaptive optics scanning laser ophthalmoscopy in fundus imaging, a review and update. Int. J. Ophthalmol. 2017, 10 (11), 1751–1758. 29181321 10.18240/ijo.2017.11.18 PMC5686376

[ref23] Weinreb R. N.; Aung T.; Medeiros F. A. The Pathophysiology and Treatment of Glaucoma: A Review. JAMA 2014, 311 (18), 1901–1911. 10.1001/jama.2014.3192. 24825645 PMC4523637

[ref24] Sepah Y. J.; Akhtar A.; Sadiq M. A.; Hafeez Y.; Nasir H.; Perez B.; Mawji N.; Dean D. J.; Ferraz D.; Nguyen Q. D. Fundus autofluorescence imaging: Fundamentals and clinical relevance. Saudi J. Ophthalmol 2014, 28 (2), 111–116. 10.1016/j.sjopt.2014.03.008. 24843303 PMC4023118

[ref25] Moreddu R.; Wolffsohn J. S.; Vigolo D.; Yetisen A. K. Laser-inscribed contact lens sensors for the detection of analytes in the tear fluid. Sens. Actuators B Chem. 2020, 317, 128183 10.1016/j.snb.2020.128183.

[ref26] Moreddu R.; Elsherif M.; Adams H.; Moschou D.; Cordeiro M. F.; Wolffsohn J. S.; Vigolo D.; Butt H.; Cooper J. M.; Yetisen A. K. Integration of paper microfluidic sensors into contact lenses for tear fluid analysis. Lab Chip 2020, 20 (21), 3970–3979. 10.1039/D0LC00438C. 32944726

[ref27] Farandos N. M.; Yetisen A. K.; Monteiro M. J.; Lowe C. R.; Yun S. H. Contact Lens Sensors in Ocular Diagnostics. Adv. Healthc. Mater. 2015, 4 (6), 792–810. 10.1002/adhm.201400504. 25400274

[ref28] Harvey D.; Hayes N. W.; Tighe B. Fibre optics sensors in tear electrolyte analysis: Towards a novel point of care potassium sensor. Cont Lens Anterior Eye 2012, 35 (3), 137–144. 10.1016/j.clae.2012.02.004. 22409950

[ref29] Hagan S.; Martin E.; Enríquez-de-Salamanca A. Tear fluid biomarkers in ocular and systemic disease: potential use for predictive, preventive and personalised medicine. EPMA J. 2016, 7 (1), 15 10.1186/s13167-016-0065-3. 27413414 PMC4942926

[ref30] Boehm D.; Keller K.; Pieter J.; Boehm N.; Wolters D.; Siggelkow W.; Lebrecht A.; Schmidt M.; Koelbl H.; Pfeiffer N.; Grus F.-H. Comparison of tear protein levels in breast cancer patients and healthy controls using a de novo proteomic approach. Oncol. Rep. 2012, 28 (2), 429 10.3892/or.2012.1849. 22664934 PMC3583517

[ref31] Csősz É.; Boross P.; Csutak A.; Berta A.; Tóth F.; Póliska S.; Török Z.; Tőzsér J. Quantitative analysis of proteins in the tear fluid of patients with diabetic retinopathy. J. Proteome Res. 2012, 75 (7), 2196–2204. 10.1016/j.jprot.2012.01.019. 22300579

[ref32] Hanyuda A.; Sawada N.; Yuki K.; Uchino M.; Ozawa Y.; Sasaki M.; Yamagishi K.; Iso H.; Tsubota K.; Tsugane S. Relationships of diabetes and hyperglycaemia with intraocular pressure in a Japanese population: the JPHC-NEXT Eye Study. Sci. Rep 2020, 10 (1), 5355–5355. 10.1038/s41598-020-62135-3. 32210291 PMC7093393

[ref33] Rentka A.; Hársfalvi J.; Berta A.; Köröskényi K.; Szekanecz Z.; Szücs G.; Szodoray P.; Kemény-Beke Á. Vascular Endothelial Growth Factor in Tear Samples of Patients with Systemic Sclerosis. Mediators Inflamm. 2015, 2015, 573681 10.1155/2015/573681. 26339137 PMC4539102

[ref34] Salvisberg C.; Tajouri N.; Hainard A.; Burkhard P. R.; Lalive P. H.; Turck N. Exploring the human tear fluid: Discovery of new biomarkers in multiple sclerosis. Prot. Clin. Appl. 2014, 8 (3–4), 185–194. 10.1002/prca.201300053. 24488530

[ref35] Tong L.; Zhou L.; Beuerman R. W.; Zhao S. Z.; Li X. R. Association of tear proteins with Meibomian gland disease and dry eye symptoms. Br. J. Ophthalmol. 2011, 95 (6), 848 10.1136/bjo.2010.185256. 21030416

[ref36] Na K.-S.; Mok J.-W.; Kim J. Y.; Rho C. R.; Joo C.-K. Correlations between Tear Cytokines, Chemokines, and Soluble Receptors and Clinical Severity of Dry Eye Disease. IOVS 2012, 53 (9), 5443–5450. 10.1167/iovs.11-9417 22789923

[ref37] López-Miguel A.; Tesón M.; Martín-Montañez V.; Enríquez-de-Salamanca A.; Stern M. E.; Calonge M.; González-García M. J. Dry Eye Exacerbation in Patients Exposed to Desiccating Stress under Controlled Environmental Conditions. Am. J. Ophthalmol. 2014, 157 (4), 788–798. 10.1016/j.ajo.2014.01.001. 24412126

[ref38] López-Miguel A.; Tesón M.; Martín-Montañez V.; Enríquez-de-Salamanca A.; Stern M. E.; González-García M. J.; Calonge M. Clinical and Molecular Inflammatory Response in Sjögren Syndrome–Associated Dry Eye Patients Under Desiccating Stress. Am. J. Ophthalmol. 2016, 161, 133–141. 10.1016/j.ajo.2015.09.039. 26456254

[ref39] Pong J. C. F.; Chu C. Y.; Li W. Y.; Tang L. Y.; Li L.; Lui W. T.; Poon T. C. W.; Rao S. K.; Lam D. S. C.; Wang C. C.; Pang C. P. Association of Hemopexin in Tear Film and Conjunctival Macrophages With Vernal Keratoconjunctivitis. Arch. Ophthalmol. 2011, 129 (4), 453–461. 10.1001/archophthalmol.2011.41. 21482871

[ref40] Leonardi A.; Palmigiano A.; Mazzola E. A.; Messina A.; Milazzo E. M. S.; Bortolotti M.; Garozzo D. Identification of human tear fluid biomarkers in vernal keratoconjunctivitis using iTRAQ quantitative proteomics. Allergy 2014, 69 (2), 254–260. 10.1111/all.12331. 24329893

[ref41] Leonardi A.; Borghesan F.; Faggian D.; Plebani M. Microarray-based IgE detection in tears of patients with vernal keratoconjunctivitis. Pediatr. Allergy Immunol. 2015, 26 (7), 641–645. 10.1111/pai.12450. 26235361

[ref42] Shetty R.; Ghosh A.; Lim R. R.; Subramani M.; Mihir K.; Reshma A. R.; Ranganath A.; Nagaraj S.; Nuijts R. M.; Beuerman R.; Shetty R.; Das D.; Chaurasia S. S.; Sinha-Roy A.; Ghosh A. Elevated expression of matrix metalloproteinase-9 and inflammatory cytokines in keratoconus patients is inhibited by cyclosporine A. Invest Ophthalmol Vis Sci. 2015, 56 (2), 738–50. 10.1167/iovs.14-14831. 25648341

[ref43] Sorkhabi R.; Ghorbanihaghjo A.; Taheri N.; Ahoor M. H. Tear film inflammatory mediators in patients with keratoconus. Int. Ophthalmol. 2015, 35 (4), 467–472. 10.1007/s10792-014-9971-3. 25062709

[ref44] Gupta D.; Wen J.; Huebner J.; Stinnett S.; Kraus V.; Tseng H. C.; Walsh M. Cytokine biomarkers in tear film for primary open-angle glaucoma. Clin Ophthalmol 2017, 11, 411–416. 10.2147/OPTH.S125364. 28260854 PMC5328319

[ref45] Rossi C.; Cicalini I.; Cufaro M. C.; Agnifili L.; Mastropasqua L.; Lanuti P.; Marchisio M.; De Laurenzi V.; Del Boccio P.; Pieragostino D. Multi-Omics Approach for Studying Tears in Treatment-Naïve Glaucoma Patients. Int. J. Mol. Sci. 2019, 20 (16), 4029 10.3390/ijms20164029. PMC6721157 31426571

[ref46] Shpak A. A.; Guekht A. B.; Druzhkova T. A.; Kozlova K. I.; Gulyaeva N. V. Brain-Derived Neurotrophic Factor in Patients with Primary Open-Angle Glaucoma and Age-related Cataract. Curr. Eye Res. 2018, 43 (2), 224–231. 10.1080/02713683.2017.1396617. 29120253

[ref47] Wang Y.; Zhao Q.; Du X. Structurally coloured contact lens sensor for point-of-care ophthalmic health monitoring. J. Mater. Chem. B 2020, 8 (16), 3519–3526. 10.1039/C9TB02389E. 31989133

[ref48] Liu Z.; Wang G.; Ye C.; Sun H.; Pei W.; Wei C.; Dai W.; Dou Z.; Sun Q.; Lin C.-T.; Wang Y.; Chen H.; Shen G. An Ultrasensitive Contact Lens Sensor Based On Self-Assembly Graphene For Continuous Intraocular Pressure Monitoring. Adv. Funct. Mater. 2021, 31 (29), 2010991 10.1002/adfm.202010991.

[ref49] Riaz R. S.; Elsherif M.; Moreddu R.; Rashid I.; Hassan M. U.; Yetisen A. K.; Butt H. Anthocyanin-Functionalized Contact Lens Sensors for Ocular pH Monitoring. ACS Omega 2019, 4 (26), 21792–21798. 10.1021/acsomega.9b02638. 31891056 PMC6933553

[ref50] Jiang N.; Montelongo Y.; Butt H.; Yetisen A. K. Microfluidic Contact Lenses. Small 2018, 14 (15), 1704363 10.1002/smll.201704363. PMC6607692 29521022

[ref51] Gabriel E. F. M.; Garcia P. T.; Cardoso T. M. G.; Lopes F. M.; Martins F. T.; Coltro W. K. T. Highly sensitive colorimetric detection of glucose and uric acid in biological fluids using chitosan-modified paper microfluidic devices. Analyst 2016, 141 (15), 4749–4756. 10.1039/C6AN00430J. 27272206

[ref52] Yetisen A. K.; Jiang N.; Castaneda Gonzalez C. M.; Erenoglu Z. I.; Dong J.; Dong X.; Stößer S.; Brischwein M.; Butt H.; Cordeiro M. F.; Jakobi M.; Hayden O.; Koch A. W. Scleral Lens Sensor for Ocular Electrolyte Analysis. Adv. Mater. 2020, 32 (6), 1906762 10.1002/adma.201906762. 31834667

[ref53] Yetisen A. K.; Jiang N.; Tamayol A.; Ruiz-Esparza G. U.; Zhang Y. S.; Medina-Pando S.; Gupta A.; Wolffsohn J. S.; Butt H.; Khademhosseini A.; Yun S.-H. Paper-based microfluidic system for tear electrolyte analysis. Lab Chip 2017, 17 (6), 1137–1148. 10.1039/C6LC01450J. 28207920 PMC5433427

[ref54] Badugu R.; Reece E. A.; Lakowicz J. R. Glucose-sensitive silicone hydrogel contact lens toward tear glucose monitoring. J. Biomed Opt 2018, 23 (5), 1–9. 10.1117/1.JBO.23.5.057005. PMC5956140 29774672

[ref55] Maejima K.; Tomikawa S.; Suzuki K.; Citterio D. Inkjet printing: an integrated and green chemical approach to microfluidic paper-based analytical devices. RSC Adv. 2013, 3 (24), 9258–9263. 10.1039/c3ra40828k.

[ref56] Yao H.; Shum A. J.; Cowan M.; Lähdesmäki I.; Parviz B. A. A contact lens with embedded sensor for monitoring tear glucose level. Biosens. Bioelectron. 2011, 26 (7), 3290–3296. 10.1016/j.bios.2010.12.042. 21257302 PMC3043144

[ref57] Kim J.; Kim M.; Lee M.-S.; Kim K.; Ji S.; Kim Y.-T.; Park J.; Na K.; Bae K.-H.; Kyun Kim H.; Bien F.; Young Lee C.; Park J.-U. Wearable smart sensor systems integrated on soft contact lenses for wireless ocular diagnostics. Nat. Commun. 2017, 8 (1), 14997 10.1038/ncomms14997. 28447604 PMC5414034

[ref58] Ku M.; Kim J.; Won J.-E.; Kang W.; Park Y.-G.; Park J.; Lee J.-H.; Cheon J.; Lee H. H.; Park J.-U. Smart, soft contact lens for wireless immunosensing of cortisol. Sci. Adv. 2020, 6 (28), eabb2891 10.1126/sciadv.abb2891. 32923592 PMC7455488

[ref59] Kim J.; Park J.; Park Y.-G.; Cha E.; Ku M.; An H. S.; Lee K.-P.; Huh M.-I.; Kim J.; Kim T.-S.; Kim D. W.; Kim H. K.; Park J.-U. A soft and transparent contact lens for the wireless quantitative monitoring of intraocular pressure. Nat. Biomed. Eng. 2021, 5 (7), 772–782. 10.1038/s41551-021-00719-8. 33941897

[ref60] Jang J.; Kim J.; Shin H.; Park Y. G.; Joo B. J.; Seo H.; Won J. E.; Kim D. W.; Lee C. Y.; Kim H. K.; Park J. U. Smart contact lens and transparent heat patch for remote monitoring and therapy of chronic ocular surface inflammation using mobiles. Sci. Adv. 2021, 7 (14), 1 10.1126/sciadv.abf7194. PMC8011975 33789904

[ref61] Elsherif M.; Hassan M. U.; Yetisen A. K.; Butt H. Wearable Contact Lens Biosensors for Continuous Glucose Monitoring Using Smartphones. ACS Nano 2018, 12 (6), 5452–5462. 10.1021/acsnano.8b00829. 29750502 PMC6107296

[ref62] VanDerMeid K. R.; Su S. P.; Ward K. W.; Zhang J.-Z. Correlation of Tear Inflammatory Cytokines and Matrix Metalloproteinases with Four Dry Eye Diagnostic Tests. IOVS 2012, 53 (3), 1512–1518. 10.1167/iovs.11-7627 22323462

[ref63] Benito M. J.; González-García M. J.; Tesón M.; García N.; Fernández I.; Calonge M.; Enríquez-de-Salamanca A. Intra- and inter-day variation of cytokines and chemokines in tears of healthy subjects. Exp. Eye Res. 2014, 120, 43–49. 10.1016/j.exer.2013.12.017. 24412438

[ref64] Tan X.; Sun S.; Liu Y.; Zhu T.; Wang K.; Ren T.; Wu Z.; Xu H.; Zhu L. Analysis of Th17-associated cytokines in tears of patients with dry eye syndrome. Eye 2014, 28 (5), 608–613. 10.1038/eye.2014.38. 24603428 PMC4017119

[ref65] Sacchetti M.; Micera A.; Lambiase A.; Speranza S.; Mantelli F.; Petrachi G.; Bonini S.; Bonini S. Tear levels of neuropeptides increase after specific allergen challenge in allergic conjunctivitis. Mol. Vis. 2011, 17, 47–52. 21245958 PMC3021574

[ref66] You J.; Hodge C.; Wen L.; McAvoy J. W.; Madigan M. C.; Sutton G. Tear levels of SFRP1 are significantly reduced in keratoconus patients. Mol. Vis. 2013, 19, 509–515. 23441124 PMC3580972

[ref67] Göncü T.; Akal A.; Adbelli F. M.; Çakmak S.; Sezen H.; Ylmaz Ö. F. Tear Film and Serum Prolidase Activity and Oxidative Stress in Patients With Keratoconus. Cornea 2015, 34 (9), 1019–1023. 10.1097/ICO.0000000000000510. 26114821

[ref68] Skwor T.; Kandel R. P.; Basravi S.; Khan A.; Sharma B.; Dean D. Characterization of Humoral Immune Responses to Chlamydial HSP60, CPAF, and CT795 in Inflammatory and Severe Trachoma. IOVS 2010, 51 (10), 5128–5136. 10.1167/iovs.09-5113 PMC3066612 20463311

[ref69] Csosz E.; Deak E.; Toth N.; Traverso C. E.; Csutak A.; Tozser J. Comparative analysis of cytokine profiles of glaucomatous tears and aqueous humour reveals potential biomarkers for trabeculectomy complications. FEBS Open Bio 2019, 9 (5), 1020–1028. 10.1002/2211-5463.12637. PMC6487689 30959565

[ref70] Naik A.; Shrivastava S.; Abidi N.; Yadav R.; Shah P.; Gala Y. Study of tear proteins for possible biomarker in primary open-angle glaucoma. JCOR 2018, 6 (2), 66–70.

[ref71] Ihnatko R.; Edén U.; Lagali N.; Dellby A.; Fagerholm P. Analysis of protein composition and protein expression in the tear fluid of patients with congenital aniridia. J. Proteome Res. 2013, 94, 78–88. 10.1016/j.jprot.2013.09.003. 24061003

[ref72] Peral A.; Carracedo G.; Pintor J. Diadenosine polyphosphates in the tears of aniridia patients. Acta Ophthalmol. 2015, 93 (5), e337–e342. 10.1111/aos.12626. 25545014

[ref73] Cicalini I.; Rossi C.; Pieragostino D.; Agnifili L.; Mastropasqua L.; di Ioia M.; De Luca G.; Onofrj M.; Federici L.; Del Boccio P. Integrated Lipidomics and Metabolomics Analysis of Tears in Multiple Sclerosis: An Insight into Diagnostic Potential of Lacrimal Fluid. Int. J. Mol. Sci. 2019, 20 (6), 1265 10.3390/ijms20061265. PMC6471885 30871169

[ref74] Kim H.-J.; Kim P.-K.; Yoo H.-S.; Kim C.-W. Comparison of tear proteins between healthy and early diabetic retinopathy patients. Clin. Biochem. 2012, 45 (1), 60–67. 10.1016/j.clinbiochem.2011.10.006. 22040812

[ref75] Costagliola C.; Romano V.; De Tollis M.; Aceto F.; dell’Omo R.; Romano M. R.; Pedicino C.; Semeraro F. TNF-Alpha Levels in Tears: A Novel Biomarker to Assess the Degree of Diabetic Retinopathy. Mediators Inflamm. 2013, 2013, 629529 10.1155/2013/629529. 24259948 PMC3821908

[ref76] Rentka A.; Harsfalvi J.; Szucs G.; Szekanecz Z.; Szodoray P.; Koroskenyi K.; Kemeny-Beke A. Membrane array and multiplex bead analysis of tear cytokines in systemic sclerosis. Immunol. Res. 2016, 64 (2), 619–626. 10.1007/s12026-015-8763-9. 26687127

[ref77] Çomoğlu S. S.; Güven H.; Acar M.; Öztürk G.; Koçer B. Tear levels of tumor necrosis factor-alpha in patients with Parkinson’s disease. Neurosci. Lett. 2013, 553, 63–67. 10.1016/j.neulet.2013.08.019. 23973333

[ref78] Badugu R.; Lakowicz J. R.; Geddes C. D. Noninvasive Continuous Monitoring of Physiological Glucose Using a Monosaccharide-Sensing Contact Lens. Anal. Chem. 2004, 76 (3), 610–618. 10.1021/ac0303721. 14750854 PMC6906081

[ref79] Wayne C. E.; Wayne R. P. Photochemistry; Oxford University Press: Oxford, 1996.

[ref80] Lu Na; S M.-h.; Pan Y.-b.; Lu W.-x.; Wu J.-l.; Li W.-y.; Zeng D.-y. Investigation on the Sub-Health Status of Healthcare Workers during the COVID-19 Pandemic. J. Infect Dis. Epidemiol. 2020, 6 (4), 152.

[ref81] Luppa P. B.; Müller C.; Schlichtiger A.; Schlebusch H. Point-of-care testing (POCT): Current techniques and future perspectives. Trends Analyt Chem. 2011, 30 (6), 887–898. 10.1016/j.trac.2011.01.019. PMC7125710 32287536

[ref82] McNaught A. D. Compendium of chemical terminology: IUPAC recommendations, 2nd ed.; Blackwell Science: Oxford, 1997.

[ref83] Lakowicz J. R. Principles of Fluorescence Spectroscopy; Boston, 2006.

[ref84] Sparks J. S.; Schelly R. C.; Smith W. L.; Davis M. P.; Tchernov D.; Pieribone V. A.; Gruber D. F. The covert world of fish biofluorescence: a phylogenetically widespread and phenotypically variable phenomenon. PLoS One 2014, 9 (1), e83259–e83259. 10.1371/journal.pone.0083259. 24421880 PMC3885428

[ref85] Penfold T. J.; Gindensperger E.; Daniel C.; Marian C. M. Spin-Vibronic Mechanism for Intersystem Crossing. Chem. Rev. 2018, 118 (15), 6975–7025. 10.1021/acs.chemrev.7b00617. 29558159

[ref86] Zhao W.; He Z.; Tang B. Z. Room-temperature phosphorescence from organic aggregates. Nat. Rev. Mater. 2020, 5 (12), 869–885. 10.1038/s41578-020-0223-z.

[ref87] Chan J.; Dodani S. C.; Chang C. J. Reaction-based small-molecule fluorescent probes for chemoselective bioimaging. Nat. Chem. 2012, 4 (12), 973–984. 10.1038/nchem.1500. 23174976 PMC4096518

[ref88] Qian H.; Cousins M. E.; Horak E. H.; Wakefield A.; Liptak M. D.; Aprahamian I. Suppression of Kasha’s rule as a mechanism for fluorescent molecular rotors and aggregation-induced emission. Nat. Chem. 2017, 9 (1), 83–87. 10.1038/nchem.2612. 27995926

[ref89] Badugu R.; Jeng B. H.; Reece E. A.; Lakowicz J. R. Contact lens to measure individual ion concentrations in tears and applications to dry eye disease. Anal. Biochem. 2018, 542, 84–94. 10.1016/j.ab.2017.11.014. 29183834 PMC5817005

[ref90] Badugu R.; Szmacinski H.; Reece E. A.; Jeng B. H.; Lakowicz J. R. Fluorescent Contact Lens for Continuous Non-invasive Measurements of Sodium and Chloride Ion Concentrations in Tears. Anal. Biochem. 2020, 608, 113902 10.1016/j.ab.2020.113902. 32800702 PMC7530058

[ref91] Mansfield J.; Gossage K.; Hoyt C.; Levenson R. Autofluorescence removal, multiplexing, and automated analysis methods for in-vivo fluorescence imaging. J. Biomed. Opt. 2005, 10 (4), 041207 10.1117/1.2032458. 16178631

[ref92] Chatterjee D. K.; Gnanasammandhan M. K.; Zhang Y. Small Upconverting Fluorescent Nanoparticles for Biomedical Applications. Small 2010, 6 (24), 2781–2795. 10.1002/smll.201000418. 21064086

[ref93] Dai N.; Kool E. T. Fluorescent DNA-based enzyme sensors. Chem. Soc. Rev. 2011, 40 (12), 5756–5770. 10.1039/c0cs00162g. 21290032 PMC5575859

[ref94] Galbán J.; Andreu Y.; Sierra J. F.; de Marcos S.; Castillo J. R. Intrinsic fluorescence of enzymes and fluorescence of chemically modified enzymes for analytical purposes: a review. Luminescence 2001, 16 (2), 199–210. 10.1002/bio.633. 11312548

[ref95] Mei J.; Leung N. L. C.; Kwok R. T. K.; Lam J. W. Y.; Tang B. Z. Aggregation-Induced Emission: Together We Shine, United We Soar. Chem. Rev. 2015, 115 (21), 11718–11940. 10.1021/acs.chemrev.5b00263. 26492387

[ref96] Framroze Z.; Conroy C. Measuring the Change in Zinc Ion Concentration in Eye Tear Fluid between Healthy and Parasite Infected Individuals: Relationship between Zinc Ions in Tear Fluid and Parasitic Infection by Soil-Transmitted Helminths. J. Med. Diagn. Methods 2016, 5 (4), 1000231.

[ref97] Gilbert R.; Peto T.; Lengyel I.; Emri E. Zinc Nutrition and Inflammation in the Aging Retina. Mol. Nutr. Food Res. 2019, 63 (15), 1801049 10.1002/mnfr.201801049. 31148351

[ref98] Chng C.-L.; Seah L. L.; Yang M.; Shen S. Y.; Koh S. K.; Gao Y.; Deng L.; Tong L.; Beuerman R. W.; Zhou L. Tear Proteins Calcium binding protein A4 (S100A4) and Prolactin Induced Protein (PIP) are Potential Biomarkers for Thyroid Eye Disease. Sci. Rep 2018, 8 (1), 16936 10.1038/s41598-018-35096-x. 30446693 PMC6240106

[ref99] Bron A. J. Diagnosis of Dry Eye. Surv. Ophthalmol. 2001, 45, S221–S226. 10.1016/S0039-6257(00)00201-0. 11587146

[ref100] Schein O. D.; Tielsch J. M.; Muñoz B.; Bandeen-Roche K.; West S. Relation between Signs and Symptoms of Dry Eye in the Elderly: A Population-based Perspective. Ophthalmology 1997, 104 (9), 1395–1401. 10.1016/S0161-6420(97)30125-0. 9307632

[ref101] Rees T. D.; LaTrenta G. S. The role of the Schirmer’s test and orbital morphology in predicting dry-eye syndrome after blepharoplasty. Plast. Reconstr. Surg. 1988, 82 (4), 619–625. 10.1097/00006534-198810000-00010. 3420183

[ref102] Bron A. J.; Evans V. E.; Smith J. A. Grading of corneal and conjunctival staining in the context of other dry eye tests. Cornea 2003, 22 (7), 640–650. 10.1097/00003226-200310000-00008. 14508260

[ref103] Korb D. R.; Greiner J. V.; Herman J. Comparison of fluorescein break-up time measurement reproducibility using standard fluorescein strips versus the Dry Eye Test (DET) method. Cornea 2001, 20 (8), 811–815. 10.1097/00003226-200111000-00007. 11685057

[ref104] GILBARD J. P.; FARRIS R. L. Ocular surface drying and tear film osmolarity in thyroid eye disease. Acta Ophthalmol. 1983, 61 (1), 108–116. 10.1111/j.1755-3768.1983.tb01401.x. 6687972

[ref105] Chotikavanich S.; de Paiva C. S.; Chen J. J.; Bian F.; Farley W. J.; Pflugfelder S. C. Production and activity of matrix metalloproteinase-9 on the ocular surface increase in dysfunctional tear syndrome. IOVS 2009, 50 (7), 3203–3209. 10.1167/iovs.08-2476 PMC3594995 19255163

[ref106] Zhou L.; Zhao S. Z.; Koh S. K.; Chen L.; Vaz C.; Tanavde V.; Li X. R.; Beuerman R. W. In-depth analysis of the human tear proteome. J. Proteome Res. 2012, 75 (13), 3877–3885. 10.1016/j.jprot.2012.04.053. 22634083

[ref107] Ruiz-Ederra J.; Levin M. H.; Verkman A. S. In Situ Fluorescence Measurement of Tear Film [Na+], [K+], [Cl−], and pH in Mice Shows Marked Hypertonicity in Aquaporin-5 Deficiency. IOVS 2009, 50 (5), 2132–2138. 10.1167/iovs.08-3033 PMC2904304 19136711

[ref108] Gartia M. R.; Misra S. K.; Ye M.; Schwartz-Duval A.; Plucinski L.; Zhou X.; Kellner D.; Labriola L. T.; Pan D. Point-of-service, quantitative analysis of ascorbic acid in aqueous humor for evaluating anterior globe integrity. Sci. Rep 2015, 5 (1), 16011 10.1038/srep16011. 26525715 PMC4630616

[ref109] Han X.-Y.; Chen Z.-H.; Fan Q.-X.; Li K.-N.; Mu F.-Y.; Luo Q.; Jin Z.; Shi G.; Zhang M. Manganese(II)-doped zinc/germanium oxide nanoparticles as a viable fluorescent probe for visual and time-resolved fluorometric determination of ascorbic acid and its oxidase. Mikrochim. Acta 2019, 186 (7), 466 10.1007/s00604-019-3580-9. 31236752

[ref110] Makaram P.; Owens D.; Aceros J. Trends in Nanomaterial-Based Non-Invasive Diabetes Sensing Technologies. Diagnostics 2014, 4 (2), 27–46. 10.3390/diagnostics4020027. 26852676 PMC4665544

[ref111] Zhu Z.; Garcia-Gancedo L.; Flewitt A. J.; Xie H.; Moussy F.; Milne W. I. A Critical Review of Glucose Biosensors Based on Carbon Nanomaterials: Carbon Nanotubes and Graphene. Sensors 2012, 12 (5), 5996–6022. 10.3390/s120505996. 22778628 PMC3386727

[ref112] Zhi Z.-L.; Khan F.; Pickup J. C. Multilayer nanoencapsulation: A nanomedicine technology for diabetes research and management. Diabetes Res. Clin. Pract. 2013, 100 (2), 162–169. 10.1016/j.diabres.2012.11.027. 23273839

[ref113] Jina A.; Tierney M. J.; Tamada J. A.; McGill S.; Desai S.; Chua B.; Chang A.; Christiansen M. Design, Development, and Evaluation of a Novel Microneedle Array-based Continuous Glucose Monitor. J. diabetes sci. technol. 2014, 8 (3), 483–487. 10.1177/1932296814526191. 24876610 PMC4455438

[ref114] Zhang W.; Du Y.; Wang M. L. On-chip highly sensitive saliva glucose sensing using multilayer films composed of single-walled carbon nanotubes, gold nanoparticles, and glucose oxidase. Sens. Bio-Sens. Res. 2015, 4, 96–102. 10.1016/j.sbsr.2015.04.006.

[ref115] Corrie S. R.; Coffey J. W.; Islam J.; Markey K. A.; Kendall M. A. F. Blood, sweat, and tears: developing clinically relevant protein biosensors for integrated body fluid analysis. Analyst 2015, 140 (13), 4350–4364. 10.1039/C5AN00464K. 25909342

[ref116] Glennon T.; O’Quigley C.; McCaul M.; Matzeu G.; Beirne S.; Wallace G. G.; Stroiescu F.; O’Mahoney N.; White P.; Diamond D. ‘SWEATCH’: A Wearable Platform for Harvesting and Analysing Sweat Sodium Content. Electroanalysis 2016, 28 (6), 1283–1289. 10.1002/elan.201600106.

[ref117] Sempionatto J. R.; Nakagawa T.; Pavinatto A.; Mensah S. T.; Imani S.; Mercier P.; Wang J. Eyeglasses based wireless electrolyte and metabolite sensor platform. Lab Chip 2017, 17 (10), 1834–1842. 10.1039/C7LC00192D. 28470263 PMC5507201

[ref118] Gao W.; Emaminejad S.; Nyein H. Y. Y.; Challa S.; Chen K.; Peck A.; Fahad H. M.; Ota H.; Shiraki H.; Kiriya D.; Lien D.-H.; Brooks G. A.; Davis R. W.; Javey A. Fully integrated wearable sensor arrays for multiplexed in situ perspiration analysis. Nature 2016, 529 (7587), 509–514. 10.1038/nature16521. 26819044 PMC4996079

[ref119] Mannoor M. S.; Tao H.; Clayton J. D.; Sengupta A.; Kaplan D. L.; Naik R. R.; Verma N.; Omenetto F. G.; McAlpine M. C. Graphene-based wireless bacteria detection on tooth enamel. Nat. Commun. 2012, 3 (1), 763 10.1038/ncomms1767. 22453836

[ref120] Senior M. Novartis signs up for Google smart lens. Nat. Biotechnol. 2014, 32, 856 10.1038/nbt0914-856. 25203024

[ref121] Ascaso F. J.; Huerva V. Noninvasive Continuous Monitoring of Tear Glucose Using Glucose-Sensing Contact Lenses. Optom Vis Sci. 2016, 93 (4), 426–434. 10.1097/OPX.0000000000000698. 26390345

[ref122] Vashist S. K. Non-invasive glucose monitoring technology in diabetes management: A review. Anal. Chim. Acta 2012, 750, 16–27. 10.1016/j.aca.2012.03.043. 23062426

[ref123] Bandodkar A. J.; Wang J. Non-invasive wearable electrochemical sensors: a review. Trends Biotechnol. 2014, 32 (7), 363–371. 10.1016/j.tibtech.2014.04.005. 24853270

[ref124] Badugu R.; Lakowicz J. R.; Geddes C. D. Ophthalmic Glucose Monitoring Using Disposable Contact Lenses—A Review. J. Fluoresc. 2004, 14 (5), 617–633. 10.1023/B:JOFL.0000039349.89929.da. 15617269 PMC4904789

[ref125] Chen C.; Dong Z.-Q.; Shen J.-H.; Chen H.-W.; Zhu Y.-H.; Zhu Z.-G. 2D Photonic Crystal Hydrogel Sensor for Tear Glucose Monitoring. ACS Omega 2018, 3 (3), 3211–3217. 10.1021/acsomega.7b02046. 31458578 PMC6641290

[ref126] Yao H.; Liao Y.; Lingley A. R.; Afanasiev A.; Lähdesmäki I.; Otis B. P.; Parviz B. A. A contact lens with integrated telecommunication circuit and sensors for wireless and continuous tear glucose monitoring. J. Micromech. Microeng. 2012, 22 (7), 075007 10.1088/0960-1317/22/7/075007.

[ref127] Zhang J.; Wang X.; Chen L.; Li J.; Luzak K. Harnessing a Nanostructured Fluorescence Energy Transfer Sensor for Quick Detection of Extremely Small Amounts of Glucose. J. diabetes Sci. Technol. 2013, 7 (1), 45–52. 10.1177/193229681300700106. 23439159 PMC3692215

[ref128] Chen L.; Tse W. H.; Chen Y.; McDonald M. W.; Melling J.; Zhang J. Nanostructured biosensor for detecting glucose in tear by applying fluorescence resonance energy transfer quenching mechanism. Biosens. Bioelectron. 2017, 91, 393–399. 10.1016/j.bios.2016.12.044. 28063388

[ref129] Badugu R.; Reece E. A.; Lakowicz J. Glucose-sensitive silicone hydrogel contact lens toward tear glucose monitoring. J. Biomed. Opt. 2018, 23 (5), 057005 10.1117/1.JBO.23.5.057005. PMC5956140 29774672

[ref130] Luo J.-J.; Pan S.-W.; Yang J.-H.; Chang T.-L.; Lin P.-Y.; Wu C.-L.; Liu W.-F.; Huang X.-R.; Koshevoy I. O.; Chou P.-T.; Ho M.-L. Detecting Glucose Levels in Blood Plasma and Artificial Tear by Au(I) Complex on the Carbopol Polymer: A Microfluidic Paper-Based Method. Polymers 2018, 10 (9), 1001 10.3390/polym10091001. PMC6404068 30960926

[ref131] Deng M.; Song G.; Zhong K.; Wang Z.; Xia X.; Tian Y. Wearable fluorescent contact lenses for monitoring glucose via a smartphone. Sens. Actuators B Chem. 2022, 352, 131067 10.1016/j.snb.2021.131067.

[ref132] Liu L.; Kong D.; Xing C.; Zhang X.; Kuang H.; Xu C. Sandwich immunoassay for lactoferrin detection in milk powder. Anal. Methods 2014, 6 (13), 4742–4745. 10.1039/C4AY00321G.

[ref133] Zhang Y.; Lu C.; Zhang J. Lactoferrin and Its Detection Methods: A Review. Nutrients 2021, 13 (8), 2492 10.3390/nu13082492. 34444652 PMC8398339

[ref134] Yamada K.; Takaki S.; Komuro N.; Suzuki K.; Citterio D. An antibody-free microfluidic paper-based analytical device for the determination of tear fluid lactoferrin by fluorescence sensitization of Tb3. Analyst (London) 2014, 139 (7), 1637–1643. 10.1039/c3an01926h. 24482793

[ref135] Yamada K.; Henares T. G.; Suzuki K.; Citterio D. Distance-Based Tear Lactoferrin Assay on Microfluidic Paper Device Using Interfacial Interactions on Surface-Modified Cellulose. ACS Appl. Mater. Interfaces 2015, 7 (44), 24864–24875. 10.1021/acsami.5b08124. 26488371

[ref136] Liu G.; Zhang K.; Nadort A.; Hutchinson M. R.; Goldys E. M. Sensitive Cytokine Assay Based on Optical Fiber Allowing Localized and Spatially Resolved Detection of Interleukin-6. ACS Sens. 2017, 2 (2), 218–226. 10.1021/acssensors.6b00619. 28723139

[ref137] Zhuang Q.-Q.; Deng H.-H.; He S.-B.; Peng H.-P.; Lin Z.; Xia X.-H.; Chen W. Immunoglobulin G-Encapsulated Gold Nanoclusters as Fluorescent Tags for Dot-Blot Immunoassays. ACS Appl. Mater. Interfaces 2019, 11 (35), 31729–31734. 10.1021/acsami.9b11599. 31411018

[ref138] Ballard Z.; Bazargan S.; Jung D.; Sathianathan S.; Clemens A.; Shir D.; Al-Hashimi S.; Ozcan A. Contact lens-based lysozyme detection in tear using a mobile sensor. Lab Chip 2020, 20 (8), 1493–1502. 10.1039/C9LC01039D. 32227027 PMC7189769

[ref139] Oliver W. T.; Wells J. E. Lysozyme as an alternative to growth promoting antibiotics in swine production. J. Anim. Sci. Biotechnol. 2015, 6 (1), 35 10.1186/s40104-015-0034-z. 26273432 PMC4535397

[ref140] Chen L.; Xia N.; Li T.; Bai Y.; Chen X. Aptasensor for visual and fluorometric determination of lysozyme based on the inner filter effect of gold nanoparticles on CdTe quantum dots. Mikrochim. Acta 2016, 183 (11), 2917–2923. 10.1007/s00604-016-1903-7.

[ref141] Chu M. X.; Miyajima K.; Takahashi D.; Arakawa T.; Sano K.; Sawada S.-i.; Kudo H.; Iwasaki Y.; Akiyoshi K.; Mochizuki M.; Mitsubayashi K. Soft contact lens biosensor for in situ monitoring of tear glucose as non-invasive blood sugar assessment. Talanta 2011, 83 (3), 960–965. 10.1016/j.talanta.2010.10.055. 21147344

[ref142] Kim S.; Jeon H.-J.; Park S.; Lee D. Y.; Chung E. Tear Glucose Measurement by Reflectance Spectrum of a Nanoparticle Embedded Contact Lens. Sci. Rep 2020, 10 (1), 8254 10.1038/s41598-020-65103-z. 32427894 PMC7237479

[ref143] Kong N.; Zhang H.; Feng C.; Liu C.; Xiao Y.; Zhang X.; Mei L.; Kim J. S.; Tao W.; Ji X. Arsenene-mediated multiple independently targeted reactive oxygen species burst for cancer therapy. Nat. Commun. 2021, 12 (1), 4777 10.1038/s41467-021-24961-5. 34362904 PMC8346549

[ref144] Parekh G.; Shi Y.; Zheng J.; Zhang X.; Leporatti S. Nano-carriers for targeted delivery and biomedical imaging enhancement. Ther. Delivery 2018, 9 (6), 451–468. 10.4155/tde-2018-0013. 29722631

[ref145] Chen L.; Hwang E.; Zhang J. Fluorescent Nanobiosensors for Sensing Glucose. Sensors 2018, 18 (5), 1440 10.3390/s18051440. PMC5982147 29734744

[ref146] Zhou M.; Zhang X.; Xie J.; Qi R.; Lu H.; Leporatti S.; Chen J.; Hu Y. pH-Sensitive Poly(β-amino ester)s Nanocarriers Facilitate the Inhibition of Drug Resistance in Breast Cancer Cells. J. Nanomater. 2018, 8 (11), 952 10.3390/nano8110952. PMC6267427 30463238

[ref147] Alam F.; Elsherif M.; AlQattan B.; Salih A.; Lee S. M.; Yetisen A. K.; Park S.; Butt H. 3D Printed Contact Lenses. ACS Biomater. Sci. Eng. 2021, 7 (2), 794–803. 10.1021/acsbiomaterials.0c01470. 33464813 PMC8396802

[ref148] Liu C.; Sun S.; Feng Q.; Wu G.; Wu Y.; Kong N.; Yu Z.; Yao J.; Zhang X.; Chen W.; Tang Z.; Xiao Y.; Huang X.; Lv A.; Yao C.; Cheng H.; Wu A.; Xie T.; Tao W. Arsenene Nanodots with Selective Killing Effects and their Low-Dose Combination with ß-Elemene for Cancer Therapy. Adv. Mater. 2021, 33 (37), 2102054 10.1002/adma.202102054. 34309925

[ref149] Yang J.; Zhang X.; Liu C.; Wang Z.; Deng L.; Feng C.; Tao W.; Xu X.; Cui W. Biologically modified nanoparticles as theranostic bionanomaterials. Prog. Mater. Sci. 2021, 118, 100768 10.1016/j.pmatsci.2020.100768.

[ref150] Moreddu R.; Vigolo D.; Yetisen A. K. Contact Lens Technology: From Fundamentals to Applications. Adv. Healthc. Mater. 2019, 8 (15), 1900368 10.1002/adhm.201900368. 31183972

[ref151] Data Bridge Market Research, Global Smart Contact Lens Market – Industry Trends and Forecast to 2029. https://www.databridgemarketresearch.com/reports/global-smart-contact-lens-market.

[ref152] La Belle J. T.; Adams A.; Lin C.-E.; Engelschall E.; Pratt B.; Cook C. B. Self-monitoring of tear glucose: the development of a tear based glucose sensor as an alternative to self-monitoring of blood glucose. ChemComm 2016, 52 (59), 9197–9204. 10.1039/c6cc03609k 27327531

[ref153] Bruen D.; Delaney C.; Florea L.; Diamond D. Glucose Sensing for Diabetes Monitoring: Recent Developments. Sensors (Basel) 2017, 17 (8), 1866 10.3390/s17081866. PMC5579887 28805693

[ref154] Luzuriaga M. A.; Berry D. R.; Reagan J. C.; Smaldone R. A.; Gassensmith J. J. Biodegradable 3D printed polymer microneedles for transdermal drug delivery. Lab Chip 2018, 18 (8), 1223–1230. 10.1039/C8LC00098K. 29536070

[ref155] Pere C. P. P.; Economidou S. N.; Lall G.; Ziraud C.; Boateng J. S.; Alexander B. D.; Lamprou D. A.; Douroumis D. 3D printed microneedles for insulin skin delivery. Int. J. Pharm. 2018, 544 (2), 425–432. 10.1016/j.ijpharm.2018.03.031. 29555437

[ref156] Krieger K. J.; Bertollo N.; Dangol M.; Sheridan J. T.; Lowery M. M.; O’Cearbhaill E. D. Simple and customizable method for fabrication of high-aspect ratio microneedle molds using low-cost 3D printing. Microsyst. Nanoeng. 2019, 5 (1), 42 10.1038/s41378-019-0088-8. 31645996 PMC6799892

[ref157] Wu M.; Zhang Y.; Huang H.; Li J.; Liu H.; Guo Z.; Xue L.; Liu S.; Lei Y. Assisted 3D printing of microneedle patches for minimally invasive glucose control in diabetes. Mater. Sci. Eng., C 2020, 117, 111299 10.1016/j.msec.2020.111299. 32919660

[ref158] Yeung C.; Chen S.; King B.; Lin H.; King K.; Akhtar F.; Diaz G.; Wang B.; Zhu J.; Sun W.; Khademhosseini A.; Emaminejad S. A 3D-printed microfluidic-enabled hollow microneedle architecture for transdermal drug delivery. Biomicrofluidics 2019, 13 (6), 064125 10.1063/1.5127778. 31832123 PMC6906119

