# Correction to “Fluorescence Sensing Technologies for Ophthalmic Diagnosis”

**DOI:** 10.1021/acssensors.4c03522

**Published:** 2025-01-10

**Authors:** Yuqi Shi, Yubing Hu, Nan Jiang, Ali K. Yetisen

**Affiliations:** † Department of Chemical Engineering, Imperial College London, South Kensington, London SW7 2BU, United Kingdom; ‡ West China School of Basic Medical Sciences and Forensic Medicine, Sichuan University, Chengdu 610041, China

The author’s correspondence has been corrected into Yuqi Shi, Yubing Hu, Nan Jiang, Ali K. Yetisen*. Ali K. Yetisen is the sole corresponding author. This change is reflected in the authorship of this Correction.

